# Endoplasmic reticulum unfolded protein response transcriptional targets of XBP-1s mediate rescue from tauopathy

**DOI:** 10.1038/s42003-024-06570-2

**Published:** 2024-07-25

**Authors:** Sarah M. Waldherr, Marina Han, Aleen D. Saxton, Taylor A. Vadset, Pamela J. McMillan, Jeanna M. Wheeler, Nicole F. Liachko, Brian C. Kraemer

**Affiliations:** 1 Geriatrics Research Education and Clinical Center, Veterans Affairs Puget Sound Health Care System, Seattle, WA 98108 USA; 2 Division of Gerontology and Geriatric Medicine, Department of Medicine, University of Washington, Seattle, WA 98104 USA; 3 Graduate Program in Neuroscience, University of Washington, Seattle, WA 98195 USA; 4 Department of Psychiatry and Behavioral Sciences, University of Washington, Seattle, WA 98195 USA; 5 Department of Pathology, University of Washington, Seattle, WA 98195 USA

**Keywords:** Alzheimer's disease, Prions, Behavioural genetics, Molecular neuroscience

## Abstract

Pathological tau disrupts protein homeostasis (proteostasis) within neurons in Alzheimer’s disease (AD) and related disorders. We previously showed constitutive activation of the endoplasmic reticulum unfolded protein response (UPR^ER^) transcription factor XBP-1s rescues tauopathy-related proteostatic disruption in a tau transgenic *Caenorhabditis elegans* (*C. elegans*) model of human tauopathy. XBP-1s promotes clearance of pathological tau, and loss of function of the ATF-6 branch of the UPR^ER^ prevents XBP-1s rescue of tauopathy in *C. elegans*. We conducted transcriptomic analysis of tau transgenic and *xbp-1s* transgenic *C. elegans* and found 116 putative target genes significantly upregulated by constitutively active XBP-1s. Among these were five candidate XBP-1s target genes with human orthologs and a previously known association with ATF6 (*csp-1*, *dnj-28*, *hsp-4*, *ckb-2*, and *lipl-3*). We examined the functional involvement of these targets in XBP-1s-mediated tauopathy suppression and found loss of function in any one of these genes completely disrupts XBP-1s suppression of tauopathy. Further, we demonstrate upregulation of HSP-4, *C. elegans* BiP, partially rescues tauopathy independent of other changes in the transcriptional network. Understanding how the UPR^ER^ modulates pathological tau accumulation will inform neurodegenerative disease mechanisms and direct further study in mammalian systems with the long-term goal of identifying therapeutic targets in human tauopathies.

## Abstract (web summary)

Genomics and transcriptomics data evaluate XBP-1s target genes conferring protection from pathological tau protein in *C. elegans*.

## Introduction

Proteins, which are the essential building blocks and catalytic machines of all cells, have a finite lifetime and can be prone to misfolding. Depending on the subcellular compartment, there are specific protein quality control mechanisms that respond to the presence of abnormal proteins, such as the cytoplasmic heat shock response, the endoplasmic reticulum (ER) unfolded protein response (UPR^ER^) with subsequent ER-associated degradation (ERAD), and the mitochondrial unfolded protein response (UPR^mt^). Once detected, multiple cytoplasmic quality control systems exist to degrade unnecessary or damaged proteins via proteolysis in order to maintain protein homeostasis (proteostasis), including the ubiquitin-proteasome system (UPS), chaperone-mediated autophagy (CMA), and macroautophagy, which will be referred to as autophagy^1^.

**Fig. 1:**
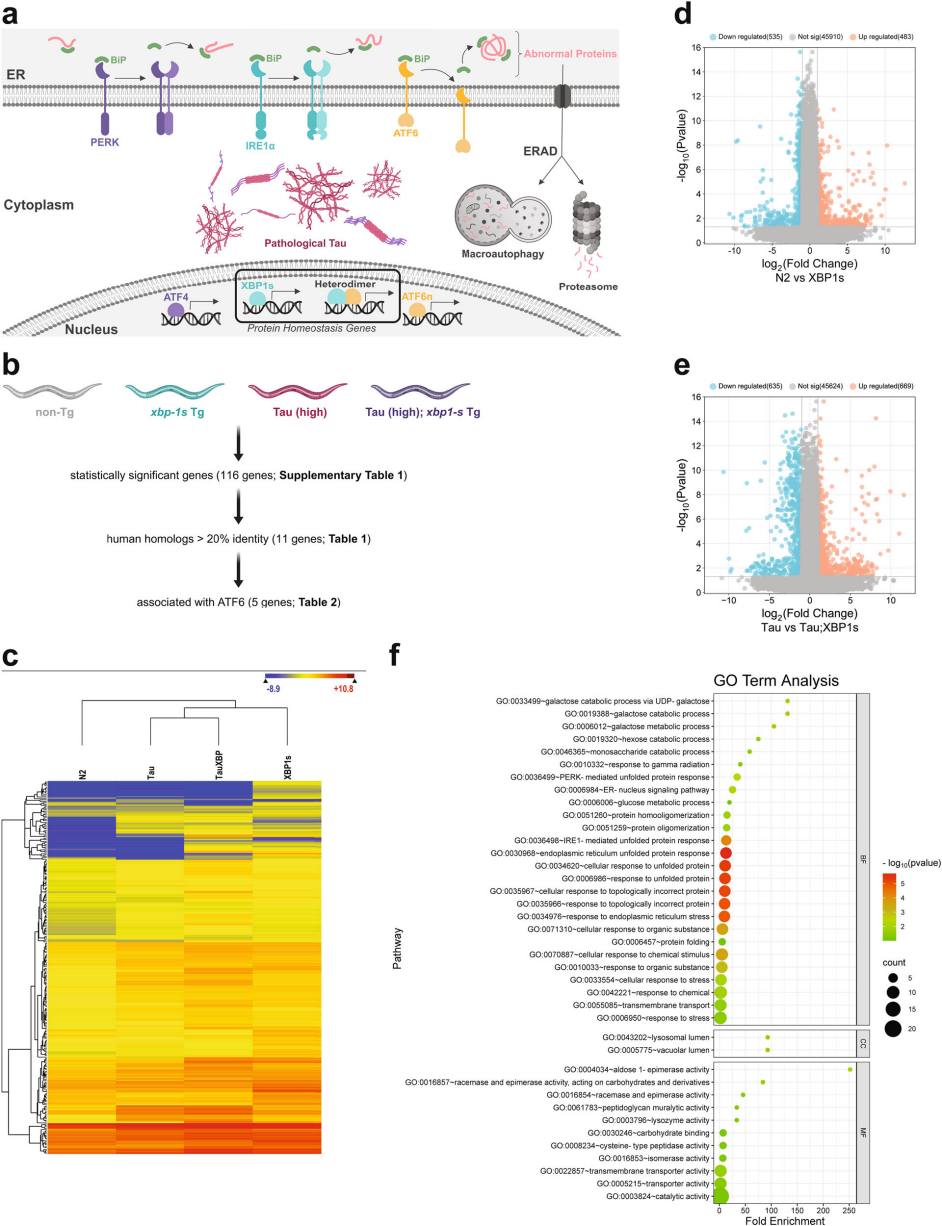
Identification of tauopathy-related XBP-1s/ATF6 target genes. **a** Schematic diagram showing subcellular compartment localization of tau in the cytoplasm, UPR^ER^ stress sensors/transducers (XBP1s, ATF6, and PERK) in the ER, and downstream transcriptional activation of protein homeostasis (proteostasis) genes in the nucleus to restore proteostasis. **b** Schematic diagram of *C. elegans* RNA sequencing workflow and comparisons. **c** Hierarchical clustering of statistically significant differentially expressed genes upregulated by the XBP-1s expressing transgene in *C. elegans*. Shown is a heatmap of target gene expression levels displayed with non-Tg (N2) as the reference gene expression level [deep blue = −8.9 differentially expressed gene (DEG) fold change, dark red = +10.8 DEG fold change]. The differentially expressed genes are upregulated and show significant changes in gene expression in all compared strains. See Supplementary Table 1 for listing of all differentially expressed genes. **d** Volcano plot of transcriptomic changes of non-Tg (N2) versus XBP-1s Tg *C. elegans* from RNA sequencing data (normalized mean read count by gene). Each point represents an individual gene. Differentially regulated genes are denoted as salmon colored highlighted data points for upregulated and teal colored highlighted data points for downregulated. **e** Volcano plot of transcriptomic changes of Tau Tg versus Tau;XBP-1s Tg *C. elegans* from RNA sequencing data (normalized mean read count by gene). Each point represents an individual gene. Differentially regulated genes are denoted as salmon colored highlighted data points for upregulated and teal colored highlighted data points for downregulated. **f** Gene Ontology (GO) analysis of candidate XBP-1s target genes for ameliorating tauopathy. These genes fall into 39 GO ID groups significantly overrepresented relative to the whole transcriptome. Schematic diagrams were created with BioRender.

Accumulation of unfolded proteins in the ER activates a transcriptional induction pathway known as the UPR^ER^ (Fig. 1a). Primary targets of the UPR^ER^ are molecular chaperones and folding enzymes localized in the ER; induction of these proteins augments the capacity of the protein folding system to restore ER proteostasis. In addition, some of the proteins involved in the ERAD system clear misfolded proteins from the ER and are upregulated by the UPR^ER^. To understand transcriptional activation of the UPR^ER^, previous work identified the *cis*-acting element and *trans*-acting factors responsible for the mammalian UPR^ER^. The *cis*-acting ER stress response element (ERSE) with the consensus sequence CCAAT-N9-CCACG is necessary and sufficient for UPR^ER^ transcriptional induction^2,3^. Because CCAAT of the ERSE is a binding site for the general transcription factor nuclear transcription factor Y (NF-Y)^3^, CCACG of the ERSE provides specificity to the UPR^ER^ in mammals^4^.

Mammalian inositol-requiring enzyme 1 α (IRE1α) is a type I ER-membrane bound endoribonuclease that initiates spliceosome-independent mRNA splicing of X-box binding protein 1 (*XBP1*) in response to ER stress^5^. The spliced form of XBP1 (XBP1s) functions as a potent transcription factor via frame switch splicing, joining the basic leucine zipper (bZIP) and transactivation domains^4^. XBP1s binds directly to the ERSE in collaboration with NF-Y to activate transcription of ER chaperone genes^4^.

Mammalian activating transcription factor 6 (ATF6) is a type II transmembrane protein in the ER activated by trans-compartmental proteolysis^6–9^. In response to ER stress, ER membrane-bound ATF6 is converted into the active transcription factor ATF6n in the Golgi apparatus, and initiates transcription of ER chaperone genes via direct binding to the ERSE in collaboration with NF-Y. ATF6n can bind to the CCACG region of the ERSE as a homodimer or heterodimer only when NF-Y is bound to the CCAAT region^10,11^.

The coordination of the three UPR^ER^ stress sensing and signaling pathways [protein kinase RNA-like ER kinase (PERK), IRE1α, and ATF6] in transcriptional remodeling has yet to be fully investigated. Previous studies indicate the activation of both the IRE1α/XBP1 and ATF6 pathways culminates in enhanced transcription at ERSE sites, thus upregulating the levels of ER chaperones^12^. The co-dependence of the IRE1α and ATF6 branches merge at XBP1: *XBP1* mRNA is induced by ATF6n and spliced by IRE1α^4^, and ATF6n heterodimerizes with XBP-1s to produce more potent transcriptional activation than XBP-1s homodimers^13^. The PERK pathway is mainly responsible for translational control, but it also plays a role in transcriptional control in mammals via induction of the transcription factor activating transcription factor 4 (ATF4). However, the ERSE is not a direct binding site of the transcription factor ATF4^14^.

Neurons are particularly vulnerable to challenges maintaining protein quality control over the lifetime. Post-mitotic neurons are more susceptible to the accumulation of cytotoxic proteins, since toxic substances cannot be diluted via cell division^15^. Throughout the course of aging, maintaining neuronal proteostasis becomes increasingly difficult. Components of the UPS, CMA, and autophagy in neurons are shown to be downregulated in both expression and activity^16^. Cytotoxicity and neuronal death resulting from misfolded oligomers and aggregates is a common molecular mechanism underlying the pathogenesis of many neurodegenerative diseases^17^.

Tau represents the most common misfolded protein in human neurodegenerative diseases^18^ and serves as a marker of brain aging. Tau proteinopathies (tauopathies) are a heterogeneous group of diseases characterized by accumulation of abnormal cytoplasmic hyperphosphorylated tau protein and associated with cognitive and motor impairments^19^. These diseases include familial frontotemporal dementia with parkinsonism linked to chromosome 17 (FTDP-17), frontotemporal lobar degeneration (FTLD-tau), argyrophilic grain disease (AGD), corticobasal degeneration (CBD), progressive supranuclear palsy (PSP), Pick’s disease (PiD), and Alzheimer’s disease (AD)^20^. AD is the most common tauopathy and cause of dementia, and its neuropathology also includes extracellular amyloid plaques composed of fibrillar amyloid beta (Aβ) peptides^21^. Mutations in the microtubule-associated protein tau (*MAPT*) gene encoding the protein tau cause FTDP-17, providing a direct link between tau dysfunction and neurodegenerative disease^22–24^. Thus far, no interventions have been found to reduce neurodegeneration or tau accumulation in these diseases.

UPR^ER^ activation has been found in postmortem brain tissue of patients with tauopathies^23^. The molecular chaperone binding immunoglobulin protein (BiP), phosphorylated PERK (pPERK), eukaryotic initiation factor 2 α (eIF2α), and IRE1α are upregulated in hippocampal neurons in AD^25,26^, while pPERK and phosphorylated IRE1α are increased in PSP, PiD, and FTLD-tau^27,28^. Of note, UPR^ER^ markers were present in neurons with diffuse phosphorylated tau, but rarely in neurons without phosphorylated tau or with aggregated phosphorylated tau, suggesting that UPR^ER^ activation occurs early on in tauopathy pathogenesis^26–28^. A recent study of late-stage AD, PiD, and PSP found no significant difference in XBP-1s, phosphorylated eIF2α, BiP, or CCAAT-enhancer-binding-protein homologous protein (CHOP) compared to non-demented controls^29^, further supporting the notion that UPR^ER^ activation is involved in the early stages of tauopathy.

Given the significance of the UPR^ER^ in aging and neurodegenerative diseases, our previous work studied genetic manipulation of the UPR^ER^ in pan-neuronal human wild type tau transgenic *C. elegans* models^30^. First, we used mild models of human wild type tau toxicity [Tau (low) transgenic *C. elegans* models]^30,31^ and found loss of function of XBP-1 and ATF-6 UPR^ER^ transcriptional branches enhanced tauopathy phenotypes, including exacerbated behavior defects, increased tau protein accumulation, and more severe neurodegeneration^30^. Next, we investigated the effects of UPR^ER^ activation in the absence of ER stress using the pan-neuronal constitutively active *xbp-1s* transgenic *C. elegans* model (*xbp-1s* Tg)^32^ and a severe model of wild type tau toxicity [Tau (high) transgenic *C. elegans* model]^30,31^. XBP-1s gain of function ameliorated tauopathy phenotypes in Tau (high) transgenic *C. elegans*, including rescued behavior defects, reduced tau accumulation, and decreased neurodegeneration, which was dependent on functional ATF-6^30^. Additionally, we showed functional ERAD via *sel-11* is required for XBP-1s-mediated tauopathy suppression in *C. elegans*^30^. Because the two main transcriptional branches of the UPR^ER^ initiate downstream activation of genes involved in ERAD, here we conducted transcriptomic analysis of XBP-1s target genes in *C. elegans* models of tauopathy. We also used putative loss-of-function mutations and gain-of-function transgenes to genetically dissect the individual roles of each target gene in XBP-1s-mediated transcriptional remodeling in tau proteostasis. Finally, we explored the translational capability of targeting the XBP1s transcriptional branch of the UPR^ER^ in mammals by generating a conditional neuronal XBP1s (niXBP1s) overexpression mouse model.

## Results

### Transcriptomic analysis reveals candidate XBP-1s target genes involved in tauopathy suppression in *C. elegans*

The IRE1α/XBP1 pathway is the most conserved branch of the UPR^ER^, leading to the expression of the master UPR^ER^ transcription factor XBP1s^33,34^. To probe UPR^ER^ function in *C. elegans* neurons, we utilized pan-neuronal *xbp-1s* gain-of-function transgenic animals (*xbp1-s* Tg) driving transcriptional activation of the XBP-1s UPR^ER^ branch in the absence of ER stress^32^. In our previous work, we demonstrated XBP-1s overexpression in neurons can rescue tauopathy in a *C. elegans* model of human tauopathy^30^. Because XBP-1s is a bZIP type transcription factor, we hypothesized XBP-1s transcriptional changes may contribute to tauopathy amelioration in *C. elegans*. To test this, we again used the pan-neuronal Tau (high) transgenic *C. elegans* model [Tau (high)], which exhibits severe behavioral phenotypes, accumulation of pathological tau protein, and neurodegeneration^30,31^. We previously demonstrated tauopathy rescue by *xbp-1s* Tg overexpression is not mediated by tau mRNA reduction, but rather changes in tau protein accumulation^30,35^. Transcriptomic analysis was conducted on young *C. elegans* at the L2 larval stage. Using the L2 larval stage as our time point for analysis provides two main advantages. First, neurodegeneration in tau transgenic animals has yet to begin^36^. Second, the full complement of neurons is present at the L2 larval stage, but other cell types such as germ cells and somatic gonad remain as progenitor cells, yielding the highest ratio of neurons to other cell types in the animal at any developmental stage^37^. We sequenced RNA from large, synchronized L2 larval stage populations of non-transgenic (non-Tg), *xbp-1s* Tg, Tau (high), and Tau (high); *xbp-1s* Tg animals using Illumina based sequencing technology. Transcriptomic analysis of the mRNA populations revealed 116 genes with robust and statistically significant expression changes (i.e., at least two-fold change in *xbp-1s* Tg versus non-Tg) (Fig. 1b–e). These candidate XBP-1s target genes for mediating tauopathy suppression are shown in Supplementary Table 1 and served as the basis for identifying regulators of tauopathy. As a control, we also analyzed tau transgene mRNA abundance and showed *xbp-1s* Tg does not alter accumulation of tau message (Supplementary Table 2), consistent with previously published observations^30,35^. Gene Ontology enrichment analysis for significantly overrepresented target genes is presented in Fig. 1f.

The three UPR^ER^ branches exert unique and coordinated transcriptional and translational remodeling to restore proteostasis, with the IRE1α/XBP1 and ATF6 branches controlling the majority of transcriptional changes. In *C. elegans*, our previous work revealed *xbp-1s*-mediated tauopathy suppression requires a functional ATF-6 branch^30^, and other studies suggest the ATF-6n transcriptional pathway might have evolved as a backup mechanism to the XBP-1s transcriptional pathway^38^. In mammals, ATF6n and XBP1s can form transcriptionally active heterodimers, which exhibit a higher binding affinity to target genes than XBP1s homodimers^13,39^. Therefore, we hypothesized the gene(s) responsible for rescue of tauopathy in *C. elegans* might be responsive to both XBP-1s and ATF-6n. To examine this possibility, we first analyzed the candidate XBP-1s target genes identified in Supplementary Table 1 for conservation between *C. elegans* and humans, revealing 11 genes (Table 1). Next, we investigated the conserved XBP1s target genes for literature evidence of ATF6n responsiveness (Table 2). From this analysis, we identified five genes exhibiting significant responsiveness to XBP-1s in the transcriptomic data that also have a previously demonstrated functional gene classification association with ATF6n: caspase 1 (*csp-1*; homologous to caspase gene family, such as CASP3, 6, 7, and 14)^40^, DNAJ domain (prokaryotic heat shock protein) 28 (*dnj-28*; homologous to DNAJC3/ p58^IPK^)^41^, heat shock protein 4 (*hsp-4*; homologous to HSP5A/BiP/GRP-78)^9,42^, choline kinase beta 2 (*ckb-2*; homologous to CHKA and CHKB)^43^, and lipase like 3 (*lipl-3*; homologous to lipase gene family, such as LIPA, LIPF, LIPJ, LIPK, LIPM, and LIPN)^44^.

**Table 1:** XBP-1s responsive genes identified by RNAseq in *C. elegans* with human orthologs

Worm gene	Human gene	Protein description (subcellular localization)	RNA fold change (*xbp-1s* Tg vs. non-Tg)
*lipl-3*	*LIP* family	Lipase (Lysosome)	18.205 ↑
*csp-1*	*CASP* family	Aspartyl Protease (Nucleus)	10.596 ↑
*dnj-28*	*DNAJC3*	DNAJ, co-chaperone of HSP70 (ER)	6.711 ↑
*F41E7.6*	*CROT*	Carnitine O-Octanoyltransferase (Peroxisome)	4.315 ↑
*erp-44.3*	*ERP44*	Protein Disulfide Isomerase (ER)	3.980 ↑
*C01B4.6*	*GALM*	Galactose Mutarotase (Cytoplasm)	3.394 ↑
*Y19D10A.16*	*GALM*	Galactose Mutarotase (Cytoplasm)	3.061 ↑
*mct-2*	*SLC16A14*	Carnitine Transporter (Plasma Membrane)	2.917 ↑
*hsp-4*	*HSPA5/BiP/GRP-78*	HSP70, chaperone (ER)	2.911 ↑
*ckb-2*	*CHKA, CHKB*	Choline Kinase (Cytoplasm)	2.151 ↑
*eol-1*	*DXO*	Decapping Exoribonuclease (Nucleus)	2.034 ↑

**Table 2:** XBP-1s responsive genes identified by RNAseq in *C. elegans* with human orthologs and ATF6 association

Worm gene	Human gene	Protein description (subcellular localization)	RNA fold change (*xbp-1s* Tg vs. non-Tg)	ATF6 association
*lipl-3*	*LIP* family	Lipase (Lysosome)	18.205 ↑	^44^
*csp-1*	*CASP* family	Aspartyl Protease (Nucleus)	10.596 ↑	^40^
*dnj-28*	*DNAJC3*	DNAJ, Co-chaperone of HSP70 (ER)	6.711 ↑	^41^
*hsp-4*	*HSPA5/BiP/GRP-78*	HSP70, chaperone (ER)	2.911 ↑	^9,42^
*ckb-2*	*CHKA, CHKB*	Choline Kinase (Cytoplasm)	2.151 ↑	^43^

### A suite of ATF6-dependent XBP-1s target genes is required for *C. elegans* tauopathy suppression

To validate the transcriptional analysis of XBP-1s responsive genes in *C. elegans* with a known association with ATF6 (Table 2), we examined available *csp-1*, *dnj-28*, *hsp-4*, *ckb-2*, and *lipl-3* putative loss-of-function mutant strains (Supplementary Table 3) in the Tau (high); *xbp-1s* Tg *C. elegans* background. We hypothesized if these five target genes are necessary, genetic loss of function might alter *xbp-1s*-mediated tauopathy suppression in *C. elegans*. Using the same strategy employed characterizing the UPR^ER^ in *C. elegans* tauopathy^30^, we first analyzed locomotion behavior, which provides a sensitive readout of neuronal function^30,31,36,45–59^. If the target gene appeared necessary for *xbp-1s*-mediated rescue of tau-induced behavioral defects, we also measured changes in total tau protein levels.

The *csp-1* gene in *C. elegans* is orthologous to the cysteine-aspartic acid protease (caspase; CASP) family of genes in humans, such as *CASP3*, *CASP6*, *CASP7*, and *CASP14*^60^. Caspases are protease enzymes playing essential roles in programmed cell death, or apoptosis. The mechanism of apoptosis is evolutionarily conserved, and the critical involvement of caspase cell death abnormality 3 (CED-3) protein in apoptosis was first discovered in *C. elegans*^61^. In mammals, 14 caspases have currently been identified and are functionally divided into three subfamilies: apoptosis initiator (CASP2, CASP8, CASP9, CASP10), apoptosis effector (CASP3, CASP6, CASP7), and inflammatory mediator (CASP1, CASP4, CASP5, CASP11, CASP12, CASP13, CASP14)^62,63^.

**Fig. 2:**
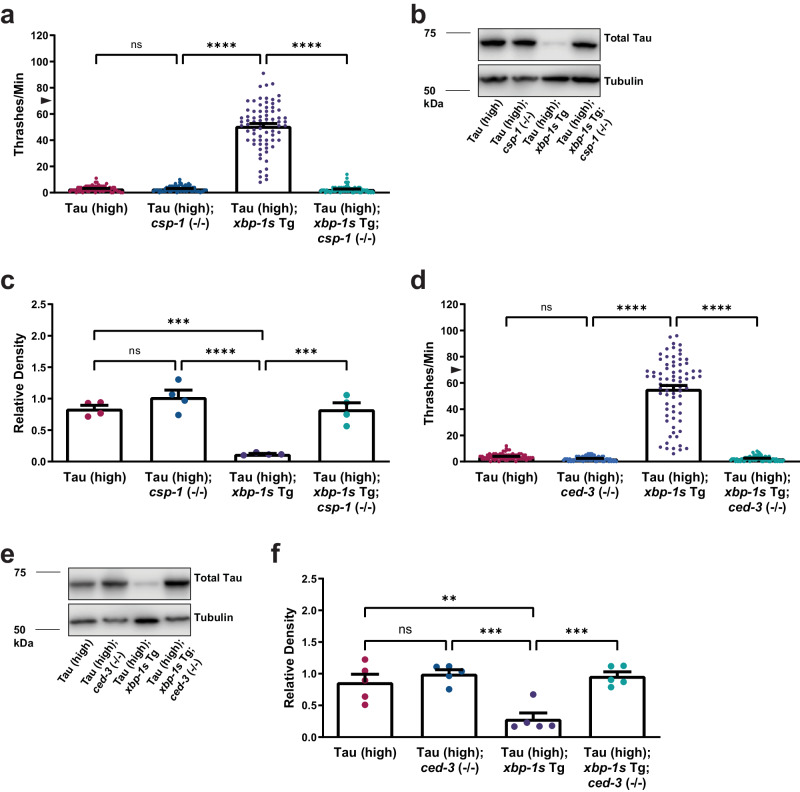
Cytoplasmic resident caspase upregulation by XBP-1s/ATF6 is required for suppression of tauopathy. Shown are genetic analyses of XBP-1s/ATF6 targets on tauopathy phenotypes in transgenic *C. elegans*. **a** *csp-1* loss of function abolishes the ability of neuronal overexpression of *xbp-1s* in Tau (high) animals to suppress severe behavioral defects observed as reflexive locomotion in response to a liquid environment [*n* = 75 animals; *N* = 5 biologically independent experiments; statistical analysis is by one-way ANOVA, followed by Tukey’s post-test (****p ≤ 0.0001)]. **b**, **c** *csp-1* loss of function abolishes the ability of neuronal overexpression of *xbp-1s* in Tau (high) animals to decrease soluble tau protein levels. Representative immunoblots for total tau (DAKO pAb) and tubulin are shown, and densitometry analysis of chemiluminescence signals for tau normalized to tubulin are plotted [*N* = 4 biologically independent experiments; statistical analysis is by one-way ANOVA, followed by Tukey’s post-test (***p ≤ 0.001, ****p ≤ 0.0001)]. **d** *ced-3* loss of function abolishes the ability of neuronal overexpression of *xbp-1s* in Tau (high) animals to suppress severe behavioral defects observed as reflexive motor impairment in response to a liquid environment [*n* = 75 animals; *N* = 5 biologically independent experiments; statistical analysis is by one-way ANOVA, followed by Tukey’s post-test (****p ≤ 0.0001)]. **e**, **f** *ced-3* loss of function abolishes the ability of neuronal overexpression of *xbp-1s* in Tau (high) animals to decrease soluble tau protein levels. Representative immunoblots for total tau (SP70 pAb) and tubulin are shown, and densitometry analysis of chemiluminescence signals for tau normalized to tubulin are plotted [*N* = 5 biologically independent experiments; statistical analysis is by one-way ANOVA, followed by Tukey’s post-test (**p ≤ 0.01, ***p ≤ 0.001)]. All bar graphs represent mean + SEM. Arrowhead on *y*-axis of all liquid thrashing bar graphs denotes non-Tg animals average ~70 thrashes/min under standard laboratory conditions. Source data are available as Supplmentary Data.

The consequence of caspase activity loss of function in *xbp-1s*-mediated tauopathy suppression was analyzed using a *C. elegans* mutant strain [*csp-1* (*ok2570*); referred to as *csp-1* (−/−)]. When compared to non-Tg animals, *csp-1* loss of function modestly enhances locomotion (Supplementary Fig. 1). We previously showed *xbp-1s* overexpression modestly induces locomotion defects when compared to non-Tg animals^30^. Tau (high); *xbp-1s* animals were crossed with *csp-1* (−/−) animals to understand the effect on tauopathy phenotypes. When we examined the deletion of *csp-1* in Tau (high) animals, we found there was no change in tau-induced behavioral dysfunction when compared to Tau (high) animals alone (Fig. 2a). As we have shown previously^30^, Tau (high); *xbp-1s* Tg animals exhibited robust rescue of the tau-mediated locomotion defects compared to Tau (high) animals alone (Fig. 2a), with a 57% rescue in motility defects compared to non-Tg^30,35^. Interestingly, *csp-1* (−/−) loss of function abolished the ability of *xbp-1s* to suppress locomotion defects as seen in Tau (high); *xbp-1s* Tg; *csp-1* (−/−) animals compared to Tau (high); *xbp-1s* Tg animals (Fig. 2a), which suggests *csp-1* upregulation by *xbp-1s* is necessary for *xbp-1s-*mediated tauopathy suppression.

Because *xbp-1s*-mediated tauopathy behavioral suppression requires *csp-1*, we also measured *csp-1* loss of function effects on total tau protein by immunoblot. Loss of function of *csp-1* did not change tau protein levels in Tau (high) animals compared to Tau (high) animals alone (Fig. 2b, c). As shown previously^30^, *xbp-1s* overexpression reduced tau protein accumulation in the Tau (high) Tg background (Fig. 2b, c), without affecting human *MAPT* transcript levels^30^ (Supplementary Table 2). Interestingly, Tau (high); *xbp-1s* Tg; *csp-1* (−/−) animals accumulated total tau protein comparable to the level seen in Tau (high) animals (Fig. 2b, c).

In *C. elegans*, there are four caspase genes (*ced-3*, *csp-1*, *csp-2*, and *csp-3*), and *csp-1* promotes programmed cell death in parallel to the canonical apoptosis pathway involving CED-3 activation^64^. To test whether the involvement of *csp-1* in *xbp-1s*-mediated tauopathy suppression is broadly applicable to overall caspase function, we crossed *ced-3* (−/−) animals [*ced-3* (*n1286*)] with Tau (high); *xbp-1s* animals. Behaviorally, *ced-3* loss of function causes locomotion defects when compared to non-Tg animals (Supplementary Fig. 1). In the Tau (high) background, *ced-3* loss of function does not affect movement defects (Fig. 2d). Surprisingly, Tau (high); *xbp-1s* Tg; *ced-3* (−/−) animals also exhibited movement defects similar to Tau (high) animals alone (Fig. 2d). The mild locomotion defects evident in *ced-3* (−/−) animals may partially block tauopathy suppression. However, we would expect the change in phenotype to be proportional to the defects in *ced-*3 (−/−) animals alone instead of the complete elimination of suppression that was observed, which suggests *ced-3* is also necessary for *xbp-1s-*mediated tauopathy suppression.

Because *xbp-1s*-mediated tauopathy behavioral suppression requires *ced-3*, we also measured *ced-3* loss of function effects on total tau protein by immunoblot. Loss of function of *ced-3* in the Tau (high) background did not alter tau protein levels when compared to Tau (high) animals alone (Fig. 2e, f). Interestingly, Tau (high); *xbp-1s* Tg; *ced-3* (−/−) animals accumulated total tau protein comparable to the level seen in Tau (high) animals (Fig. 2e, f). Taken together, these data demonstrate overall caspase activity via either CSP-1 or CED-3 is necessary for *xbp-1s* gain of function to suppress behavioral and biochemical tauopathy phenotypes observed in the *C. elegans* Tau (high) background.

*C. elegans dnj-28* is orthologous to human *DNAJC3*, which encodes the ER stress-regulated chaperone DNAJ heat shock protein family (Hsp40) member C3 (DNAJC3), also known as 58-kilodalton inhibitor of protein kinase (P58^IPK^). ER stress induces the transcription of *DNAJC3*^65^. DNAJC3 has diverse functions in the ER, including being a co-chaperone and regulator of BiP^66–69^ as well as inhibiting PERK, thereby relieving the PERK-mediated translational attenuation^65^.

**Fig. 3:**
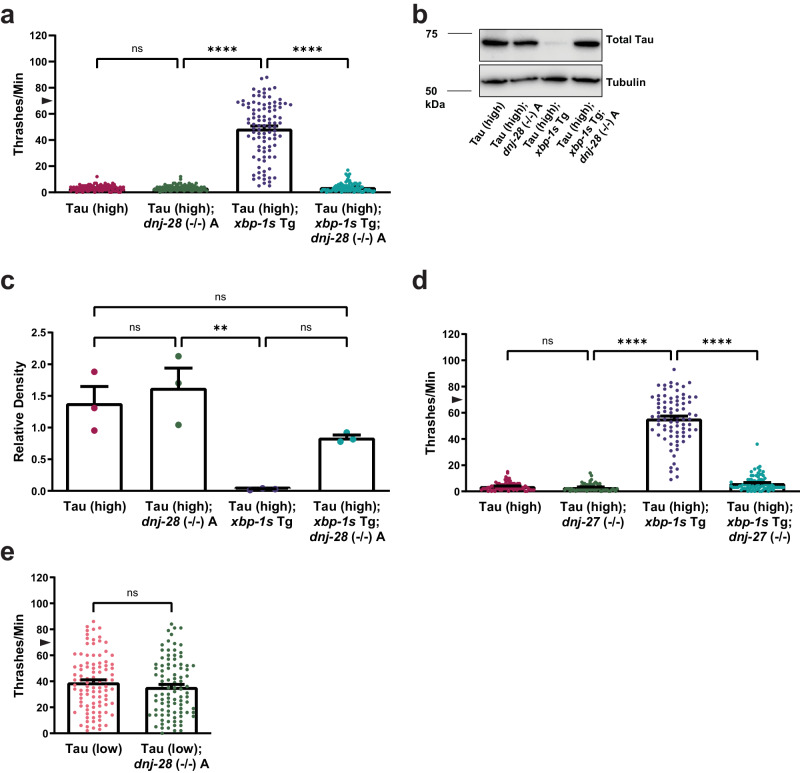
ER resident co-chaperone upregulation by XBP-1s/ATF6 is required for suppression of tauopathy. Shown are genetic analysis of XBP-1s/ATF6n targets on tauopathy phenotypes in transgenic *C. elegans*. **a** *dnj-28* loss of function abolishes the ability of neuronal overexpression of *xbp-1s* in Tau (high) animals to suppress severe behavioral defects observed as reflexive motor impairment in response to a liquid environment [*n* = 97 animals; *N* = 7 biologically independent experiments; statistical analysis is by one-way ANOVA, followed by Tukey’s post-test (****p ≤ 0.0001)]. **b**, **c** *dnj-28* loss of function trends toward but does not significantly reduce soluble tau protein levels caused by neuronal *xbp-1s* overexpression in Tau (high) animals. Representative immunoblots for total tau (DAKO pAb) and tubulin are shown, and densitometry analysis of chemiluminescence signals for tau normalized to tubulin are plotted [*N* = 3 biologically independent experiments; statistical analysis is by one-way ANOVA, followed by Tukey’s post-test (**p ≤ 0.01)]. **d** *dnj-27* loss of function abolishes the ability of neuronal overexpression of *xbp-1s* in Tau (high) animals to suppress severe behavioral defects observed as reflexive motor impairment in response to a liquid environment [*n* = 80, 74, 80, 80 animals, respectively; *N* = 4 biologically independent experiments; statistical analysis is by one-way ANOVA, followed by Tukey’s post-test (****p ≤ 0.0001)]. **e** *dnj-28* loss of function in Tau (low) animals does not alter mild behavioral defects observed as reflexive motor impairment in response to a liquid environment [*n* = 96 and 94 animals, respectively; *N* = 5 biologically independent experiments; statistical analysis is by unpaired *t*-test, two tailed (ns: *p* = 0.2586)]. All bar graphs represent mean + SEM. Arrowhead on *y*-axis of all liquid thrashing bar graphs denotes non-Tg animals average ~70 thrashes/min under standard laboratory conditions. Source data are available as Supplmentary Data.

We investigated the effects of *dnj-28* loss of function in *xbp-1s*-mediated tauopathy suppression in *C. elegans* by using a putative loss-of-function mutant strain [*dnj-28* (*ok2490*); referred to as *dnj-28* (−/−) A] crossed to Tau (high); *xbp-1s* Tg animals. Compared to non-Tg animals, there is no significant difference in movement with *dnj-28* loss of function (Supplementary Fig. 2). Deletion of *dnj-28* in Tau (high) animals did not change the tau-induced locomotion abnormalities when compared to Tau (high) animals alone (Fig. 3a). However, *dnj-28* loss of function prevented the ability of *xbp-1s* Tg to suppress behavioral defects in Tau (high); *xbp-1s* Tg; *dnj-28* (−/−) A animals when compared to Tau (high); *xbp-1s* Tg animals (Fig. 3a). To validate these findings, we generated an additional independent molecular null allele in *dnj-28* where the entire coding sequence of *dnj-28* was deleted [*dnj-28* (*bk3074*); referred to as *dnj-28* (−/−) B]. Analysis of *dnj-28* (−/−) B replicated the findings in *dnj-28* (−/−) A (Supplementary Fig. 3).

Because *dnj-28* is required for *xbp-1s*-mediated tauopathy behavioral suppression, we also measured the effects of *dnj-28* loss of function on total tau protein by immunoblot. Loss of function of *dnj-28* alone did not change tau protein levels in Tau (high) animals compared to Tau (high) animals alone (Fig. 3b, c). Interestingly, Tau (high); *xbp-1s* Tg; *dnj-28* (−/−) A animals accumulated total tau protein comparable to the level seen in Tau (high) animals (Fig. 3b, c).

The DNAJ domain class of genes in *C. elegans* includes 29 genes^60^. To test whether the requirement of functional *dnj-28* for *xbp-1s*-mediated tauopathy suppression is broadly applicable to other DNAJ domain genes, we chose to investigate *dnj-27* loss of function [*dnj-27* (*ok2302*); referred to as *dnj-27* (−/−)]. Downregulation of *dnj-27* expression by RNAi in human Aβ, α-synuclein, and polyglutamine (polyQ) transgenic *C. elegans* models enhances neurodegenerative phenotypes^70^. In the Tau (high) background, *dnj-27* loss of function does not affect movement defects (Fig. 3d). Surprisingly, Tau (high); *xbp-1s* Tg; *dnj-27* (−/–) animals also exhibited movement defects similar to Tau (high) alone animals (Fig. 3d). Therefore, two DNAJ domain genes (*dnj-28* and *dnj-27*) are both required for *xbp-1s*-mediated tauopathy suppression in *C. elegans*.

In addition, we probed the importance of *dnj-28* in modulating tauopathy. Because the severity of behavioral dysfunction of Tau (high) animals might affect our ability to discriminate enhancement of tauopathy phenotypes, we used the pan-neuronal Tau (low) transgenic *C. elegans* model, which exhibits mild behavioral deficits and no significant accumulation of pathological tau species^30,31^. If *dnj-28* regulates tau proteostasis in *C. elegans*, we expected Tau (low) animals crossed with the *dnj-28* loss-of-function mutant strain would exhibit enhancement of tau-dependent locomotion dysfunction. However, when crossed with Tau (low) animals, *dnj-28* (−/−) A did not worsen Tau (low) behavioral defects (Fig. 3e), demonstrating that *dnj-28* loss of function does not exacerbate tauopathy in the absence of overt UPR^ER^ induction by *xbp-1s* overexpression.

The *C. elegans* gene *hsp-4* is an ortholog of the human gene heat shock protein A5 (*HSPA5*), which encodes the UPR^ER^ sensor protein BiP. As one of the most abundant proteins in the ER, BiP maintains ER homeostasis via a variety of functions, including protein folding processes, protein import into the ER, regulation of calcium homeostasis, and facilitation of ERAD^71^. Under ER stress conditions, BiP can initiate the UPR^ER^, decrease unfolded protein load, induce autophagy, and crosstalk with apoptosis machinery to assist in the cell survival decision^71^.

**Fig. 4:**
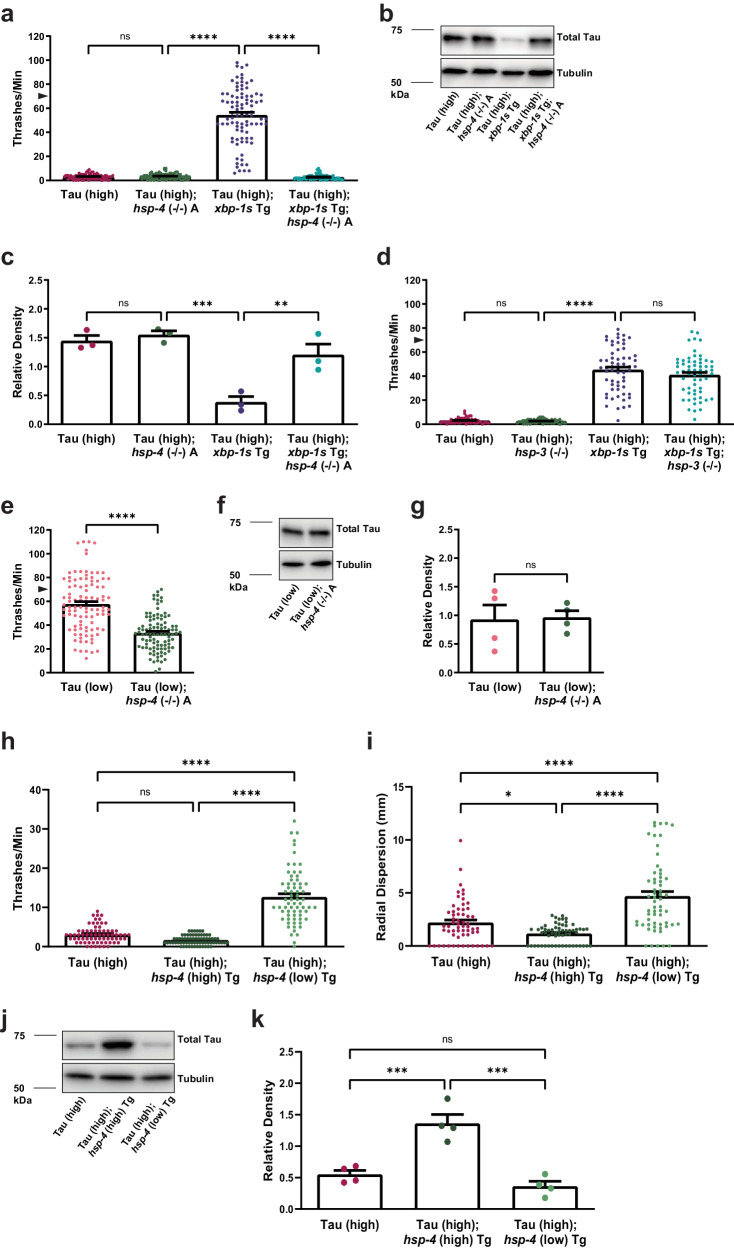
ER resident chaperone HSP-4/BiP upregulation by XBP-1s/ATF6 is required for suppression of tauopathy. Shown are genetic analyses of XBP-1s/ATF6 targets on tauopathy phenotypes in transgenic *C. elegans*. **a** *hsp-4* loss of function abolishes the ability of neuronal overexpression of *xbp-1s* in Tau (high) animals to suppress severe behavioral defects observed as reflexive motor impairment in response to a liquid environment [*n* = 90 animals; *N* = 6 biologically independent experiments; statistical analysis is by one-way ANOVA, followed by Tukey’s post-test (****p ≤ 0.0001)]. **b**, **c** *hsp-4* loss of function abolishes the ability of neuronal overexpression of *xbp-1s* in Tau (high) animals to decrease soluble tau protein levels. Representative immunoblots for total tau (SP70 pAb) and tubulin are shown, and densitometry analysis of chemiluminescence signals for tau normalized to tubulin are plotted [*N* = 3 biologically independent experiments; statistical analysis is by one-way ANOVA, followed by Tukey’s post-test (**p ≤ 0.01, ***p ≤ 0.001)]. **d** *hsp-3* loss of function does not alter the ability of neuronal overexpression of *xbp-1s* in Tau (high) animals to suppress severe behavioral defects observed as reflexive motor impairment in response to a liquid environment [*n* = 60 animals; *N* = 4 biologically independent experiments; statistical analysis is by one-way ANOVA, followed by Tukey’s post-test (****p ≤ 0.0001)]. **e** *hsp-4* loss of function in Tau (low) animals enhances mild behavioral defects observed as reflexive motor impairment in response to a liquid environment [*n* = 100 animals; *N* = 5 biologically independent experiments; statistical analysis is by unpaired *t*-test, two tailed (****p ≤ 0.0001)]. **f**, **g** *hsp-4* loss of function in Tau (low) animals does not affect soluble tau protein levels. Representative immunoblots for total tau (SP70 pAb) and tubulin are shown, and densitometry analysis of chemiluminescence signals for tau normalized to tubulin are plotted [*N* = 4 biologically independent experiments; statistical analysis is by unpaired *t*-test, two-tailed (ns: *p* = 0.9003)]. **h** Neuronal high overexpression of *hsp-4* in Tau (high) animals does not alter severe behavioral defects, while neuronal low overexpression of *hsp-4* in Tau (high) animals mildly suppresses severe behavioral defects observed as reflexive motor impairment in response to a liquid environment [*n* = 70, 70, and 69 animals, respectively; *N* = 5 biologically independent experiments; statistical analysis is by one-way ANOVA, followed by Tukey’s post-test (****p ≤ 0.0001)]. **i** Neuronal h**i**gh overexpression of *hsp-4* in Tau (high) animals enhances severe behavioral defects, while neuronal low overexpression of *hsp-4* in Tau (high) animals mildly suppresses severe behavioral defects observed in an unstimulated environment [*n* = 60 animals; *N* = 3 biologically independent experiments; statistical analysis is by one-way ANOVA, followed by Tukey’s post-test (****p ≤ 0.0001)]. **j**, **k** Neuronal high overexpression of *hsp-4* in Tau (high) animals increases soluble tau protein levels, while neuronal low overexpression of *hsp-4* in Tau (high) animals does not affect soluble tau protein levels. Representative immunoblots for total tau (DAKO pAb) and tubulin are shown, and densitometry analysis of chemiluminescence signals for tau normalized to tubulin are plotted [*N* = 4 biologically independent experiments; statistical analysis is by one-way ANOVA, followed by Tukey’s post-test (***p ≤ 0.001)]. All bar graphs represent mean + SEM. Arrowhead on *y*-axis of all liquid thrashing bar graphs denotes non-Tg animals average ~70 thrashes/min under standard laboratory conditions. Source data are available as Supplmentary Data.

To understand the potential role of BiP in *xbp-1s*-mediated tauopathy suppression in *C. elegans*, we used an *hsp-4* loss-of-function mutant strain [*hsp-4* (*gk514*); referred to as *hsp-4* (−/−) A]. When compared to non-Tg animals, *hsp-4* (−/−) A animals had mild locomotion defects (Supplementary Fig. 4). Deletion of *hsp-4* in Tau (high) animals did not affect tau-induced behavioral dysfunction when compared to Tau (high) animals alone (Fig. 4a). However, *hsp-4* loss of function completely eliminated *xbp-1s-*mediated rescue of behavioral phenotype in Tau (high); *xbp-1s* Tg; *hsp-4* (−/−) A animals (Fig. 4a). The mild locomotion defects evident in *hsp-4* (−/−) A animals may partially block tauopathy suppression. Although we would expect the change in phenotype to be proportional to the defects in *hsp-4* (−/−) A animals alone instead of the complete reversal of suppression that was observed, which suggests *hsp-4* upregulation by *xbp-1s* is necessary for *xbp-1s-*mediated tauopathy suppression. To validate these findings, we generated an additional independent molecular null allele in *hsp-4* where the entire coding sequence of *hsp-4* was deleted [*hsp-4* (*bk3060*); referred to as *hsp-4* (−/−) B]. Analysis of *hsp-4* (−/−) B replicated the findings in *hsp-4* (−/−) A (Supplementary Fig. 5).

Because *xbp-1s*-mediated tauopathy behavioral suppression requires *hsp-4*, we also measured *hsp-4* loss of function effects on total tau protein by immunoblot. Loss of function of *hsp-4* alone did not change tau protein levels in Tau (high) animals compared to Tau (high) animals alone (Fig. 4b, c). Interestingly, Tau (high); *xbp-1s* Tg; *hsp-4* (−/−) A animals accumulated total tau protein comparable to the level seen in Tau (high) animals (Fig. 4b, c). Taken together, these data demonstrate *hsp-4* loss of function significantly impacts the ability of *xbp-1s* gain of function to suppress behavioral and biochemical tauopathy phenotypes observed in the *C. elegans* Tau (high) background.

In *C. elegans*, HSP-3 and HSP-4 are homologous to mammalian BiP. HSP-3 is both constitutively expressed and stress-responsive, while HSP-4 has very low basal expression in most cells, but is strongly induced by UPR^ER^ signaling^72,73^. Although *hsp-4* was responsive to *xbp-1s* upregulation in our transcriptomics data, *hsp-3* was not. Tau (high); *xbp-1s* Tg animals were crossed with a *C. elegans hsp-3* mutant strain [*hsp-3* (*ok1083*); referred to as *hsp-3* (−/−)]. When compared to non-Tg animals, *hsp-3* (−/−) animals had mild locomotion defects (Supplementary Fig. 2). Tau (high); *hsp-3* (−/−) animals displayed a similar defect in locomotion when compared to Tau (high) animals alone (Fig. 4d). When compared to Tau (high); *xbp-1s* Tg animals, Tau (high); *xbp-1s* Tg; *hsp-3* (−/−) animals exhibited similar suppression of tauopathy-induced locomotion defects (Fig. 4d), indicating *hsp-3* loss of function is dispensable for *xbp-1s*-mediated tauopathy suppression. Although HSP-3 and HSP-4 proteins are highly conserved and thought to be functionally redundant^73^, we show *xbp-1s*-mediated tauopathy suppression in *C. elegans* is specific, requiring functional HSP-4.

Given the lack of *hsp-3* involvement, we further explored the impact of HSP-4 function on modulating tauopathy phenotypes in the absence of overt UPR^ER^ induction by *xbp-1s* overexpression in neurons. Because of the severity of behavioral dysfunction of Tau (high) animals, we again used Tau (low) animals. We observed Tau (low); *hsp-4* (−/−) A animals displayed significant enhancement of the locomotion defects seen in Tau (low) Tg animals alone (Fig. 4e). We further probed *hsp-4* involvement in tau proteostasis and found no significant increase in total tau species detected by immunoblot in Tau (low); *hsp-4* (−/−) A animals compared to Tau (low) animals (Fig. 4f, g). Additionally, we observed no change in phosphorylated tau in Tau (low); *hsp-4* (−/−) animals (Supplementary Fig. 6).

Because HSP-4 loss of function blocks *xbp-1s*-mediated tauopathy suppression of behavioral and biochemical phenotypes in the Tau (high) background and enhances tauopathy phenotypes in the Tau (low) background, we hypothesized *hsp-4* gain of function alone might play a role in tauopathy in *C. elegans*. We generated pan-neuronal *hsp-4* transgenic *C. elegans* lines with varying expression levels (Supplementary Fig. 7) using a different neuronal promoter than those driving Tau (high) and *xbp-1s* Tg *C. elegans* expression. Both neuronal low overexpression [*hsp-4* (low) Tg] and high overexpression [*hsp-4* (high) Tg] *C. elegans* models were generated and have limited or no impact on health and motor function (Supplementary Figs. 8 and 9). We crossed *hsp-4* Tg animals with Tau (high) animals and analyzed the effect on tau-induced locomotion defects. Interestingly, when compared to Tau (high) animals alone, Tau (high); *hsp-4* (high) Tg animals exhibited similar tau-induced behavioral defects as Tau (high) animals (Fig. 4h). However, low overexpression of *hsp-4* was able to mildly suppress behavioral tauopathy phenotypes, as seen in Tau (high); *hsp-4* (low) Tg animals compared to Tau (high) animals alone (Fig. 4h). To confirm the effect of *hsp-4* overexpression levels on tauopathy movement phenotypes, we used the radial locomotion assay, which measures movement in the absence of an external stimulus. Interestingly, there was a significant reduction in radial dispersion of Tau (high); *hsp-4* (high) Tg animals compared to Tau (high) animals alone, while *hsp-4* low overexpression modestly increased radial dispersion in the Tau (high) background (Fig. 4i). This confirms our other behavioral results for *hsp-4* low overexpression, with this assay also able to discriminate the movement defect with *hsp-4* high overexpression in the Tau (high) background (Fig. 4h). We also examined the consequence of *hsp-4* overexpression on tau proteostasis by immunoblot and found high overexpression of *hsp-4* in the Tau (high) background significantly increases total tau species (Fig. 4j, k). However, low overexpression of *hsp-4* in the Tau (high) background does not significantly alter total tau protein levels when compared to Tau (high) animals (Fig. 4j, k). These results indicate HSP-4 function can mediate tauopathy suppression in *C. elegans*, but requires a fine-tuned expression level.

**Fig. 5:**
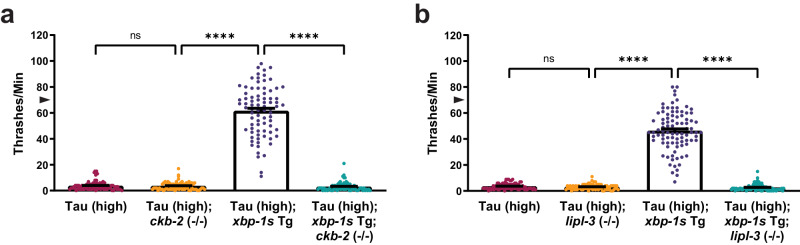
Cytoplasmic resident choline kinase CKB-2 and lysosomal resident lipase LIPL-3 upregulation by XBP-1s/ATF6 is required for suppression of tauopathy. Shown are genetic analyses of XBP-1s/ATF6 targets on tauopathy phenotypes in transgenic *C. elegans*. **a** *ckb-2* loss of function abolishes the ability of neuronal overexpression of *xbp-1s* in Tau (high) animals to suppress severe behavioral defects observed as reflexive motor impairment in response to a liquid environment [*n* = 80 animals; *N* = 4 biologically independent experiments; statistical analysis is by one-way ANOVA, followed by Tukey’s post-test (****p ≤ 0.0001)]. **b** *lipl-3* loss of function abolishes the ability of neuronal overexpression of *xbp-1s* in Tau (high) animals to suppress severe behavioral defects observed as reflexive motor impairment in response to a liquid environment [*n* = 90 animals; *N* = 6 biologically independent experiments; statistical analysis is by one-way ANOVA, followed by Tukey’s post-test (****p ≤ 0.0001)]. All bar graphs represent mean + SEM. Arrowhead on *y*-axis of all liquid thrashing bar graphs denotes non-Tg animals average ~70 thrashes/min under standard laboratory conditions. Source data are available as Supplmentary Data.

The *C. elegans* gene *ckb-2* is an ortholog of human choline kinase alpha (CHKA) and choline kinase beta (CHKB). These proteins participate in the cytidine diphosphocholine (CDP-choline) pathway to synthesize phosphatidylcholine for ER biogenesis, which is upregulated by both ATF6α^43^ and XBP-1s^74^. The *ckb-2* loss of function [*ckb-2* (*bk3106*); referred to as *ckb-2* (−/−)] in Tau (high) animals did not change tau-induced motor deficits. However, deletion of *ckb-2* in Tau (high); *xbp-1s* Tg animals eliminated the ability of *xbp-1s* to suppress tauopathy, indicating intact *ckb-2* is required for *xbp-1s* to exert its suppressive effect (Fig. 5a).

The *lipl-3* gene in *C. elegans* is orthologous to a human class of genes known as lipase (LIP) family members, including *LIPA*, *LIPF, LIPJ*, *LIPK*, *LIPM*, and *LIPN*^60^. The *LIPA* gene exhibits the highest homology to *lipl-3* and encodes the lysosomal acid lipase (LAL) enzyme found in lysosomal compartments, which is essential for lipid metabolism. LAL breaks down lipids such as cholesterol esters and triglycerides to generate free fatty acids and cholesterol^75^. To understand whether functional LIPL-3 is necessary for *xbp-1s*-mediated tauopathy suppression, we crossed Tau (high); *xbp-1s* Tg animals with a loss-of-function mutant strain [*lipl-3* (*gk846191*); referred to as *lipl-3* (−/−)] and explored the effect on tauopathy locomotion defects. Compared to non-Tg animals, there is no significant difference in movement with *lipl-3* loss of function (Supplementary Fig. 2). Loss of function of *lipl-3* did not significantly affect Tau (high) locomotion defects (Fig. 5b). In contrast, Tau (high); *xbp-1s* Tg; *lipl-3* (−/−) animals displayed similar locomotion abnormalities when compared to Tau (high) animals alone (Fig. 5b), indicating *lipl-3* is also required for *xbp-1s*-mediated tauopathy suppression in *C. elegans*.

Taken together, five genes identified as responsive to XBP-1s in the transcriptomic data with a previously known association with ATF6n (*csp-1*, *dnj-28*, *hsp-4*, *ckb-2*, and *lipl-3*; Table 2) are all essential for *xbp-1s*-mediated tauopathy suppression in *C. elegans*. However, in absence of overt UPR^ER^ induction by *xbp-1s* overexpression in neurons, these target genes differentially affect tauopathy phenotypes, which warrants future investigation.

### A suite of other XBP-1s target genes is also required for *C. elegans* tauopathy suppression

To understand whether XBP-1s responsive genes in *C. elegans* rely on an association with ATF6 (Table 2), we also examined the remaining six *C. elegans* genes with human homologs, but no previous association with ATF6 (Table 1). We utilized available *erp-44.3*, *F41E7.6*, *C01B4.6*, *Y19D10A.16*, *eol-1*, and *mct-2* putative loss-of-function mutant strains (Supplementary Table 3) in the Tau (high); *xbp-1s* Tg *C. elegans* background. We hypothesized if these target genes are also necessary for tauopathy suppression, their genetic loss of function would also block *xbp-1s*-mediated tauopathy suppression in *C. elegans*.

**Fig. 6:**
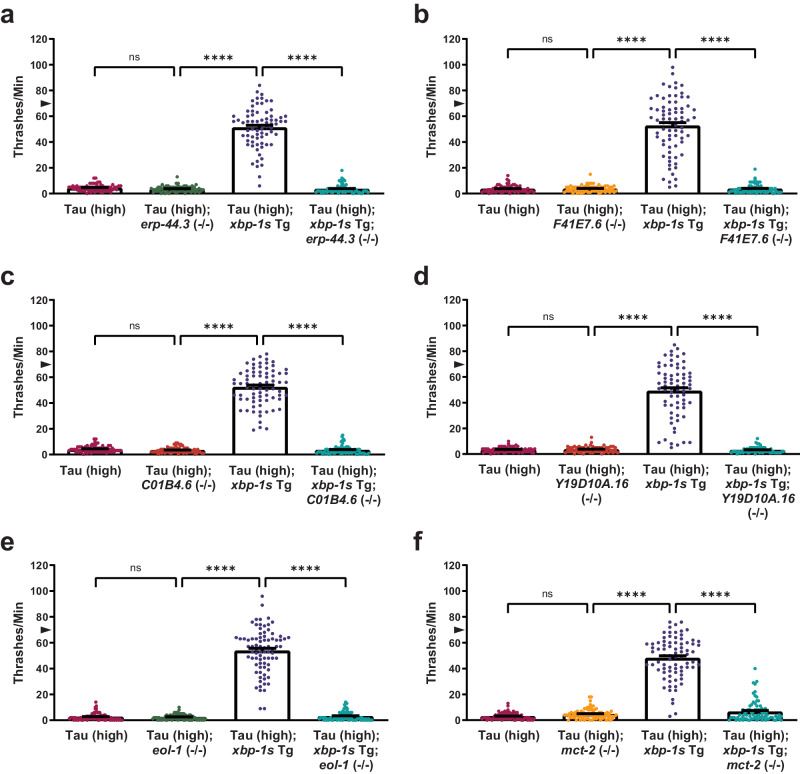
Additional XBP-1s target genes are required for suppression of tauopathy. Shown are genetic analyses of XBP-1s targets with human homologs lacking a known association with ATF6 on tauopathy phenotypes in transgenic *C. elegans*. **a** *erp-44.3* loss of function abolishes the ability of neuronal overexpression of *xbp-1s* in Tau (high) animals to suppress severe behavioral defects observed as reflexive motor impairment in response to a liquid environment [*n* = 75 animals; *N* = 5 biologically independent experiments; statistical analysis is by one-way ANOVA, followed by Tukey’s post-test (****p ≤ 0.0001)]. **b** *F41E7.6* loss of function abolishes the ability of neuronal overexpression of *xbp-1s* in Tau (high) animals to suppress severe behavioral defects observed as reflexive motor impairment in response to a liquid environment [*n* = 75 animals; *N* = 5 biologically independent experiments; statistical analysis is by one-way ANOVA, followed by Tukey’s post-test (****p ≤ 0.0001)]. **c** *C01B4.6* loss of function abolishes the ability of neuronal overexpression of *xbp-1s* in Tau (high) animals to suppress severe behavioral defects observed as reflexive motor impairment in response to a liquid environment [*n* = 75 animals; *N* = 5 biologically independent experiments; statistical analysis is by one-way ANOVA, followed by Tukey’s post-test (****p ≤ 0.0001)]. **d** *Y19D10A.16* loss of function abolishes the ability of neuronal overexpression of *xbp-1s* in Tau (high) animals to suppress severe behavioral defects observed as reflexive motor impairment in response to a liquid environment [*n* = 70 animals; *N* = 5 biologically independent experiments; statistical analysis is by one-way ANOVA, followed by Tukey’s post-test (****p ≤ 0.0001)]. **e** *eol-1* loss of function abolishes the ability of neuronal overexpression of *xbp-1s* in Tau (high) animals to suppress severe behavioral defects observed as reflexive motor impairment in response to a liquid environment [*n* = 80 animals; *N* = 4 biologically independent experiments; statistical analysis is by one-way ANOVA, followed by Tukey’s post-test (****p ≤ 0.0001)]. **f** *mct-2* loss of function abolishes the ability of neuronal overexpression of *xbp-1s* in Tau (high) animals to suppress severe behavioral defects observed as reflexive motor impairment in response to a liquid environment [*n* = 85, 87, 79, and 77 animals, respectively; *N* = 5 biologically independent experiments; statistical analysis is by one-way ANOVA, followed by Tukey’s post-test (****p ≤ 0.0001)]. All bar graphs represent mean + SEM. Arrowhead on *y*-axis of all liquid thrashing bar graphs denotes non-Tg animals average ~70 thrashes/min under standard laboratory conditions. Source data are available as Supplementary Data.

The *C. elegans* gene *erp-44.3* is orthologous to human endoplasmic reticulum protein 44 (*ERP44*), which encodes a pH-regulated chaperone in the protein disulfide isomerase (PDI) family. ERP44 retrieves mislocalized or incompletely assembled proteins from the ER-Golgi intermediate compartment or cis-Golgi and deposits them back in the ER, thereby acting as a quality control mechanism for secreted proteins^76^. Compared to non-Tg animals, there is no significant difference in movement with *erp-44.3* loss of function [*erp-44.3* (*tm6492*); referred to as *erp-44.3* (−/−)] (Supplementary Fig. 2). By crossing *erp-44.3* (−/−) into the Tau (high); *xbp-1s* Tg background, we showed loss of function of *erp-44.3* blocked *xbp-1s*-mediated suppression of tau-induced locomotion deficits, while *erp-44.3* loss of function in the Tau (high) background did not change locomotion compared to Tau (high) animals alone (Fig. 6a), indicating *erp-44.3* is also required for *xbp-1s*-mediated tauopathy suppression.

The *C. elegans* gene *F41E7.6* is an ortholog of human *CROT* and is predicted to encode a carnitine O-octanoyltransferase (CROT) in the carnitine acyltransferase family of enzymes. CROT catalyzes the transfer of medium- and long-chain fatty acyl groups from coenzyme A (CoA) to L-carnitine to facilitate transport of these molecules out of the peroxisome and into the cytosol and mitochondria for further fatty acid metabolism^77^. When compared to non-Tg animals, *F41E7.6* (−/−) animals [*F41E7.6* (*tm5587*)] had mild locomotion defects (Supplementary Fig. 2). Loss of function of *F41E7.6* in the Tau (high) background did not alter tau-induced locomotion abnormalities when compared to Tau (high) animals alone (Fig. 6b). However, when *F41E7.6* (−/−) was crossed to Tau (high); *xbp-1s* Tg animals, *xbp-1s* overexpression failed to suppress tau-induced behavioral defects, indicating that *F41E7.6* was also necessary for this mechanism (Fig. 6b).

*C01B4.6* is a *C. elegans* ortholog of the human gene *GALM*, which encodes a galactose mutarotase (GALM), also known as aldose 1-epimerase. This enzyme converts β- to α-D-galactose (and, to a much lesser extent, glucose) for downstream metabolism^78^. When compared to non-Tg animals, *C01B4.6* (−/−) animals [*C01B4.6* (*tm6913*)] had mild locomotion defects (Supplementary Fig. 2). Loss of function of *C01B4.6* in Tau (high) animals had no effect on tau-induced behavioral deficits compared to Tau (high) animals alone (Fig. 6c). However, crossing *C01B4.6* (−/−) with Tau (high); *xbp-1s* Tg animals resulted in loss of *xbp-1s*-mediated suppression of tau-induced locomotion impairment (Fig. 6c). *Y19D10A.16* is another *C. elegans* ortholog of the human gene *GALM*. When compared to non-Tg animals, *Y19D10A.16* (−/−) animals [*Y19D10A.16* (*bk3109*)] had mild locomotion defects (Supplementary Fig. 2). Deletion of this gene in Tau (high) animals resulted in behavioral deficits similar to Tau (high) alone (Fig. 6d). However, loss of *Y19D10A.16* reversed the behavioral rescue seen in Tau (high); *xbp-1s* Tg animals (Fig. 6d), indicating both *GALM* orthologs (*C01B4.6*, *Y19D10A.16*) are also necessary for *xbp-1s* to suppress tau-induced motor impairment.

The *C. elegans* gene *eol-1* is orthologous to the human gene *DXO*, which encodes a decapping exoribonuclease (DXO) with pyrophosphohydrolase, decapping, and 5′-3′ exoribonuclease activity^79^. DXO contributes to RNA capping quality control by removing incomplete 5’ N7-methylguanosine (m7G) or non-canonical nicotinamide adenine dinucleotide (NAD), flavin adenine dinucleotide (FAD), and dephospho-CoA caps from RNA^80,81^. To investigate whether *eol-1* is required for *xbp-1s*-mediated tauopathy suppression in *C. elegans*, we used an *eol-1* loss-of-function mutant strain [*eol-1* (*gk534833*); referred to as *eol-1* (−/−)] crossed to Tau (high); *xbp-1s* Tg animals. When compared to non-Tg animals, *eol-1* (−/−) animals had mild locomotion defects (Supplementary Fig. 2). Loss of function of *eol-1* in Tau (high) animals had no effect on tau-induced locomotion impairment when compared to Tau (high) animals alone (Fig. 6e). However, *eol-1* loss of function blocked the ability of *xbp-1s* Tg to suppress tau-induced behavioral defects in Tau (high); *xbp-1s* Tg; *eol-1* (−/−) animals when compared to Tau (high); *xbp-1s* Tg animals (Fig. 6e), which indicates *eol-1* is also required for *xbp-1s*- mediated suppression of tauopathy.

The *C. elegans* gene *mct-2* is orthologous to human *SLC16A4* in the monocarboxylate transporter family, also known as the solute carrier 16 (SLC16) family^82–84^. Its protein is predicted to transport monocarboxylic acids such as pyruvate, lactate, and ketone bodies across the plasma membrane. When compared to non-Tg animals, *mct-2* loss of function [*mct-2* (*bk3105*); referred to as *mct-2* (−/−)] modestly enhances locomotion (Supplementary Fig. 2). While deletion of *mct-2* does not affect tau-induced motility defects in Tau (high) animals, it blocks suppression of these defects in Tau (high); *xbp-1s* Tg animals (Fig. 6f). Therefore, *xbp-1s* also requires functional *mct-2* to suppress tauopathy phenotype.

Taken together, many *C. elegans* genes with human homologs (Table 1), which function in disparate molecular pathways, are required for *xbp-1s* overexpression to suppress tau-induced behavioral deficits in *C. elegans*. In addition to the five genes identified with a previously known association with ATF6 (*csp-1*, *dnj-28*, *hsp-4*, *ckb-2*, and *lipl-3*; Table 2), each of the six XBP-1s target genes without previous association with ATF6 (*erp-44.3*, *F41E7.6*, *C01B4.6*, *Y19D10A.16*, *eol-1*, and *mct-2*; Table 1) must be intact for XBP-1s-mediated suppression of tauopathy in *C. elegans*. These results indicate ATF-6 association may not be necessary for determining which downstream targets of XBP-1s are required to ameliorate disease phenotype. Furthermore, these results support the robustness of the XBP-1s transcriptional analysis in *C. elegans* and provide information on potential mediators of tauopathy.

### XBP1s induction upregulates tauopathy-related target genes, but causes lethality in mice

**Fig. 7:**
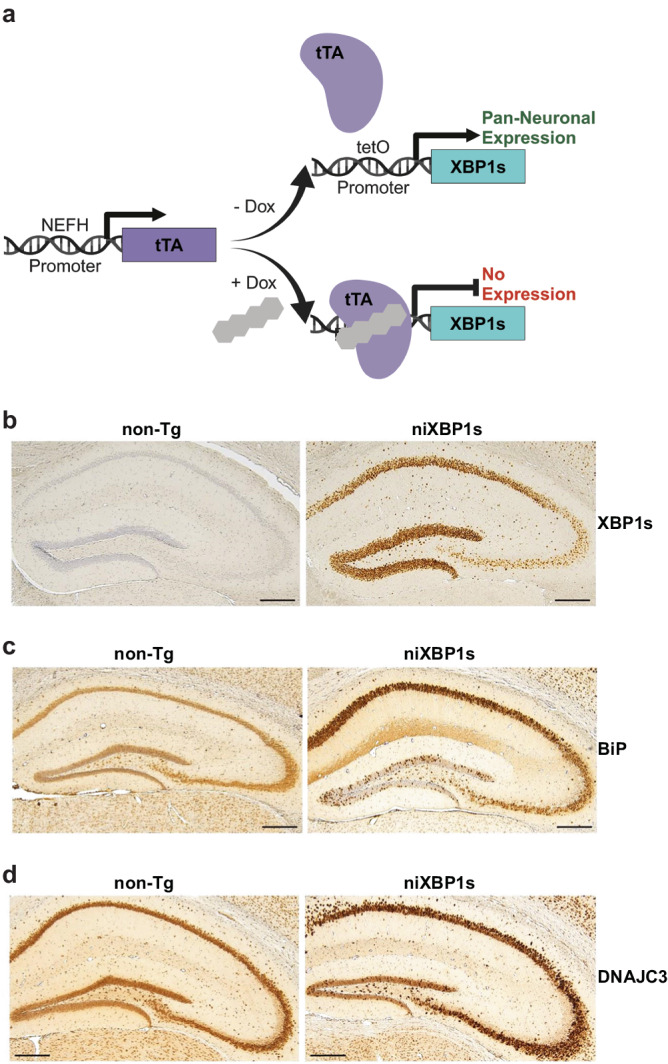
niXBP1s activation in mouse brain upregulates tauopathy-related target genes. **a** Schematic diagram of Dox-regulatable pan-neuronal expression of XBP1s in bigenic mice under the control of the *NEFH* promoter. **b** XBP1s induction is detectable by immunohistochemistry in niXBP1s mice off Dox. Representative hippocampal brain sections from 4-month-old non-Tg and niXBP1s mice stained with XBP1s antibody are shown. **c** BiP, the mouse homolog of *C. elegans hsp-4*, is upregulated in niXBP1s mice off Dox. Representative hippocampal brain sections from 4-month-old non-Tg and niXBP1s mice stained with BiP/HSPA5 antibody are shown. **d** DNAJC3, the mouse homolog of *C. elegans dnj-28*, is upregulated in niXBP1s mice off Dox. Representative hippocampal brain sections from 4-month-old non-Tg and niXBP1s mice stained with DNAJC3 antibody are shown. Scale bars: 500 µm. Schematic diagram was created with BioRender.

To assess the translational relevance of UPR^ER^ hyperactivation in the mammalian brain, we generated a mouse model conditionally overexpressing mouse XBP1s specifically in neurons. To produce these mice, we employed the tetracycline reverse transcriptional activator (rtTA)-driven conditional XBP1s transgene previously generated^85^ as a responder transgene and crossed them to the previously generated neurofilament heavy chain (NEFH) promoter driver transgene^86,87^ to yield double transgenic neuron-inducible XBP1s (niXBP1s) mice (Fig. 7a). Double transgenic mice appear viable and healthy when transgene expression is suppressed by tetracycline. Young adult niXBP1s mice exhibit abundant XBP1s protein readily detectable in hippocampal neurons by 10 days after transgene induction (Fig. 7b). We evaluated a subset of the mouse homologs of candidate *C. elegans xbp-1s* target genes identified above and observed HSPA5/BiP, the mouse homolog of *hsp-4*, becomes dramatically upregulated in hippocampal neurons in niXBP1s mice, confirming conservation of *hsp-4*/BiP as an XBP-1s target gene across phyla (Fig. 7c). Similarly, DNAJC3, the mouse homolog of *C. elegans xbp-1s* target gene *dnj-28*, becomes induced in the hippocampus of niXBP1s mice (Fig. 7d). While initially healthy, niXBP1s mice exhibit a sudden death phenotype between 2–3 weeks post-induction, precluding the long-term evaluation of niXBP1s impacts on aging-related tauopathy phenotypes. We suspect this early death phenotype is driven by sustained UPR^ER^ activation and subsequent ER stress-induced apoptosis through the CHOP pathway^88,89^. We attribute the long-term tolerability of XBP-1s expression in *C. elegans* neurons to the absence of a homolog of CHOP in the *C. elegans* genome^60^.

## Discussion

Aging-related declines in cellular function and neurodegenerative diseases are closely associated with overall cellular proteostasis, including the UPR^ER^. Much work has been conducted to understand the role of the UPR^ER^ in the context of health and disease over the lifespan. However, contradictory results from studies in neurodegenerative tauopathies warrants a more direct approach to dissect the underlying mechanisms of the UPR^ER^ in tauopathy. Because the three stress transducers of the UPR^ER^ each control transcriptional regulation via XBP1s, ATF6n, and ATF4 transcription factors, understanding the role of downstream upregulated target genes is important for identifying unique regulators of tauopathy. Here, we followed up on our previous study^30^ by exploring the role of UPR^ER^ transcriptional regulation via XBP-1s in human wild type tau transgenic *C. elegans*.

Constitutive activation of the master UPR^ER^ transcription factor XBP-1s suppresses tauopathy phenotypes in *C. elegans*, which relies on functional ATF-6^30^. Because XBP-1s is essential for UPR^ER^ transcriptional remodeling, we conducted whole organism transcriptomic analysis at the L2 developmental stage to enrich for neuronal transcripts upregulated by XBP-1s in Tau (high) *C. elegans*. We narrowed our search to find candidate XBP-1s target genes in three sequential steps: identifying significant genes upregulated in *xbp-1s* Tg animals compared to non-Tg animals (116 genes; Supplementary Table 1), identifying significant genes with human homologs (11 genes; Table 1), and identifying significant genes with human homologs and a known association with ATF6 (5 genes; Table 2). From this analysis, we nominated multiple genetic modifiers of tauopathy associated with XBP-1s/ATF6 in *C. elegans*, including *csp-1*, *dnj-28*, *hsp-4*, *ckb-2*, and *lipl-3*.

Another study conducted intestine-specific RNA sequencing analysis in *C. elegans* to identify genes upregulated in the intestine when XBP-1s is overexpressed in neurons through non-cell autonomous UPR^ER^ signaling^90^. Neuronal XBP-1s significantly increased the expression of genes involved in lysosome function, and intestinal lysosome function was necessary for enhanced lifespan and proteostasis. Compared to genes in Table 1, *dnj-28*, *erp-44.3*, *hsp-4*, and *ckb-2* were also upregulated in this RNA sequencing dataset. Consistent with our results regarding the lipase family member *lipl-3* gene, *lipl-1*, *lipl-2*, and *lipl-5* were also significantly upregulated in the intestine in response to neuronal *xbp-1s* Tg overexpression. However, *csp-1*, *F41E7.6*, *C01B4.6*, *Y19D10A.16*, *mct-2*, and *eol-1* were not identified as significantly upregulated or downregulated. These differences indicate a potential divergence of neuronal XBP-1s in tauopathy suppression versus lifespan extension, which warrants further investigation.

To genetically dissect the contributions of each XBP-1s/ATF6 candidate gene, we systematically eliminated the function of *csp-1*, *dnj-28*, *hsp-4*, *ckb-2*, and *lipl-3* in Tau (high); *xbp-1s* Tg *C. elegans*. We supported the validity of the transcriptomic analysis by showing putative loss of function of five candidate genes abolished *xbp-1s*-mediated tauopathy suppression in *C. elegans* (Figs. 2–5). To fully understand the specificity of these nominated genes in *C. elegans* tauopathy, we tested whether another member of the same functional gene classification affected Tau (high); *xbp-1s* Tg animal phenotypes. In contrast to *hsp-4* loss of function (Fig. 4a and Supplementary Fig. 5), *hsp-3* (−/−) did not affect *xbp-1s*-mediated behavioral tauopathy suppression (Fig. 4d). Interestingly, *ced-3* (Fig. 2d) and *dnj-27* (Fig. 3d) are also required for tauopathy suppression via *xbp-1s*, indicating a potential broader role of caspase and DNAJ domain genes in *xbp-1s*-mediated tauopathy suppression.

Additionally, we tested whether the nominated XBP-1s target genes could modulate tauopathy in *C. elegans* in the absence of *xbp-1s*-mediated UPR^ER^ induction. We show *hsp-4* loss of function enhanced behavioral tauopathy phenotypes without affecting total tau protein (Fig. 4e–g), while *dnj-28* loss of function does not regulate tauopathy in Tau (low) animals (Fig. 3e). We also generated neuronal *hsp-4* overexpression transgenic *C. elegans* models and crossed these with Tau (high) animals. We show low expression of *hsp-4* modestly suppressed tauopathy behavioral phenotypes, while *hsp-4* high expression had no effect or modestly enhanced behavioral phenotypes (Fig. 4h, i). Interestingly, *hsp-4* low overexpression did not affect total tau protein, while *hsp-4* high overexpression increased total tau protein in the Tau (high) background (Fig. 4j, k). Given the intricate nature of XBP-1s-mediated transcriptional remodeling, future work should explore loss of function and gain of function of all candidate target genes in tauopathy suppression.

**Fig. 8:**
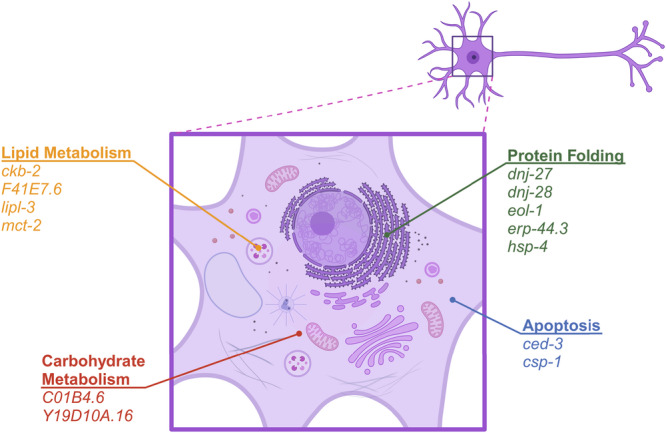
XBP-1s transcriptional remodeling requires concerted upregulation of a suite of genes for tauopathy suppression. Schematic diagram showing the 13 genes comprising four components of the *xbp-1s* transcriptional network and subcellular localization required for tauopathy suppression in *C. elegans*: protein folding (*dnj-27*, *dnj-28*, *eol-1*, *erp-44.3*, and *hsp-4*), lipid metabolism (*ckb-2*, *F41E7.6*, *lipl-3*, and *mct-2*), carbohydrate metabolism (*C01B4.6* and *Y19D10A.16*), and apoptosis (*ced-3* and *csp-1*). Schematic diagram was created with BioRender.

Finally, we also investigated putative loss of function of XBP-1s candidate genes with human homologs, but without previous association with ATF6: *erp-44.3*, *F41E7.6* (*CROT* ortholog), *C01B4.6* (*GALM* ortholog), *Y19D10A.16* (*GALM* ortholog), *eol-1*, and *mct-2*. Surprisingly, *xbp-1s*-mediated suppression of tauopathy also requires each of these genes (Fig. 6). Given their varied subcellular localization and functions, *xbp-1s* activates a diverse and non-redundant network of genes required for tauopathy suppression in *C. elegans* (Table 1 and Fig. 8).

The first component of the proposed *xbp-1s* transcriptional network involves protein folding target genes. The most apparent network members participate in protein processing capacity in the UPR^ER^ and include *hsp-4*, *dnj-28*, *dnj-27*, and *erp-44.3*. The *C. elegans* homolog of BiP, HSP-4, senses misfolded proteins and initiates the UPR^ER^. Consequently, *hsp-4* low overexpression improves tau-induced locomotor defects in the Tau (high) *C. elegans* model (Fig. 4h, i), presumably by acting downstream of UPR^ER^ activation. DNAJC3, the mammalian homolog of DNJ-28*8*, can recruit BiP to the ER membrane and help degrade misfolded proteins. It also increases ER protein folding capacity by inhibiting PERK, which downregulates translation^65^. DNAJC3 knockout in mice results in an inability to cope with ER stress, and in humans causes multisystemic neurodegeneration and diabetes mellitus^91^, suggesting an integral role in ER and cellular processing of proteins. Since ERP44 acts as a sentry for mislocalized enzymes and disulfide-linked oligomeric proteins between the ER and Golgi, we would expect its upregulation to enhance cellular capacity for protein quality control. Altogether, these three genes work directly to improve proteostasis in the cell.

As a decapping exoribonuclease, *eol-1* appears to represent a distinct pathway downstream of *xbp-1s*. However, mRNA cap-independent translation has been demonstrated to be a feature of UPR^ER^-mediated gene expression changes in UPR^ER^-regulated fatty acid biosynthesis^92^. Further, regulation of *C. elegans hsp-4* and cell death genes has been demonstrated to occur through a cap-independent mechanism^93^. Thus, *eol-1* could indirectly support the functions of chaperones by enhancing their translation in the face of ER stress. The action of *eol-1* may also ameliorate the UPR^ER^ by degrading mRNAs encoding secretory proteins targeted to the ER, thereby reducing protein load while enriching for ER resident chaperones to enhance the UPR^ER^ capacity to clear misfolded proteins.

We propose a second component of the *xbp-1s* transcriptional network for lipid metabolism involving *lipl-3*, *mct-2*, *F41E7.6* (*CROT* ortholog), and *ckb-2*. These genes may be involved in *xbp-1s*-induced lipid remodeling, which increases longevity and protects against proteotoxic stress and accumulation of misfolded protein species in *C. elegans*^94,95^. This lipid gene expression remodeling may also reflect *xbp-1s*-induced ER expansion^74^, which would involve *ckb-2*, or the UPR^ER^ transcriptional response to lipid bilayer stress^96^.

The third component of the proposed *xbp-1s* transcriptional network involves carbohydrate metabolism. Our transcriptomic dataset yielded two *C. elegans* homologs of *GALM* (*C01B4.6*, *Y19D10A.16*), providing convincing evidence this gene is indeed upregulated in response to *xbp-1s* overexpression. While involvement of GALM in the UPR^ER^ remains unexplored, this enzyme is upstream of uridine diphosphate (UDP)-galactose 4-epimerase in the Leloir pathway, which converts galactose to glucose. In mammals, XBP1s regulates UDP-galactose 4-epimerase expression in the liver for protein glycosylation under fed conditions^85^. Therefore, an increased expression of GALM may reflect an upregulation of the Leloir pathway in *xbp-1s* Tg *C. elegans* through a transcriptional program distinct from pathological UPR^ER^ activation and represent signal from non-neuronal cells as a result of whole-animal RNA sequencing^97^ or a *C. elegans*-specific pathway. Indeed, non-cell autonomous signaling by XBP-1s between neurons and the intestine in *C. elegans* promotes stress resistance and longevity^32,98^, so it seems unsurprising to detect the downstream transcriptional effects of neuronal XBP-1s overexpression in peripheral tissues.

Lastly, we propose a fourth component of the *xbp-1s* transcriptional network for apoptosis involving *csp-1* and *ced-3*, which are homologous to the human *CASP* family of genes. *C. elegans* possess four caspases: CED-3, CSP-1, CSP-2, and CSP-3. Of these, CED-3 is an essential *C. elegans* caspase required for cell death^99^. However, further work has shown CSP-1B can cleave the CED-3 proprotein in vitro, suggesting *C. elegans* apoptosis may involve a proteolytic cascade^100^, and CSP-1B can kill cells mostly independent of CED-3^64^. In mammals, CASP3, 6, and 7 are considered the executioners in apoptosis^101^. Chronic ER stress upregulates CASP3^102^ and induces apoptosis^103^. CASP6 and CASP6-cleaved tau are found in tangles and neurites of AD frontal and temporal cortex^104^, while immunodetection of CASP3 is limited to hippocampal neurons undergoing granulovacuolar degeneration^105^. Both CASP3 and 6 colocalize with hyperphosphorylated tau in AD brainstem^106^.

The absolute requirement for each one of the major XBP-1s target genes for tauopathy suppression in *C. elegans* is truly surprising. While the necessity of inducible BiP expression as a component of canonical UPR^ER^ function was fully anticipated, the same level of involvement for each of the other XBP-1s target genes (Table 1 and Figs. 2–6) was not an expected outcome. We do not currently have a conceptual framework for the UPR^ER^ transcriptional network that adequately explains these observations. On the surface, we take these observations as support for a hitherto undescribed molecular feedback mechanism for the UPR^ER^ whereby XBP-1s target genes reinforce UPR^ER^-mediated tauopathy rescue through a transcriptional program. However, the molecular mechanism for such a diverse set of proteins to all mediate UPR^ER^ transcriptional activity does not seem readily apparent. Neither does downstream involvement of all Table 1 target genes seem likely to be directly linked to tau proteostasis, as we show for *dnj-28* loss of function in the Tau (low) background (Fig. 3e). Thus, we plan to continue the molecular investigation of this phenomena through a classical forward genetic approach in *C. elegans* beyond the scope of this transcriptomic study.

The *C. elegans* model system provides many advantages for dissecting the underlying mechanisms of pathology in human neurodegenerative diseases that are not easily achieved in intact mammalian model systems. However, to understand the translational capability of UPR^ER^ activation in mammals, we generated niXBP1s mice (Fig. 7a). We found robust expression of XBP1s and two target gene proteins (BiP and DNAJC3) in hippocampal neurons of niXBP1s mice (Fig. 7b–d). Another group also recently generated a conditional and neuron-inducible XBP1s transgenic mouse model using a different driver transgene promoter, calcium/calmodulin dependent protein kinase II alpha (CAMK2A)^107^. Consistent with the sudden death phenotype observed in niXBP1s mice, these mice exhibited progressively worsening seizures followed by death approximately 2 weeks after induction of *Xbp1s* overexpression^107^.

In contrast to these unexpected sudden death phenotypes resulting from sustained overexpression of XBP1s in neurons, prior XBP1s overexpression studies in mice showed overall beneficial effects on brain function. One group generated a transgenic mouse model which constitutively expresses moderate levels of XBP1s under the control of the prion promoter^108^. These mice exhibited improved basal learning capacity associated with improved long-term potentiation (LTP) and synaptic transmission in the hippocampus^108^. In a follow-up study, this group also found XBP1s overexpression reduced Aβ deposition and preserved synaptic and cognitive function in the AD mouse model expressing five familial AD mutations (5xFAD)^109^. Additionally, local delivery of XBP1s via adeno-associated vectors into the hippocampus of 5xFAD mice after Aβ has begun accumulating also restored cognitive function and synaptic plasticity^109^. Another group used *Xbp1s*-expressing viral vectors delivered into the hippocampus of triple transgenic AD model mice (3xTg-AD; a less aggressive AD model compared to 5xFAD)^110^. In this AD mouse model, XBP1s overexpression prevented the loss of dendritic spines and improved neuronal plasticity^110^. Differences in the degree of expression, sustained versus transient overexpression, or cell type specificity of expression related to gene promoters used could explain the contrasting phenotypes observed among these different XBP1s mouse models. Regardless, we believe the absence of a CHOP encoding gene in the *C. elegans* genome underlies the lack of toxic phenotype by prolonged *xbp-1s* activation in *C. elegans* neurons and the presence of CHOP drives the dose-dependent toxicity in mice discussed above.

Recently, it was shown ER chaperones act downstream of the transcription factor nuclear receptor subfamily 4, group A, member 1 (Nr4a1) to effect changes in synaptic plasticity required for long-term memory in mice^111^. Blocking Nr4a1 transcriptional activity resulted in downregulation of pathways related to ER protein processing, chaperone binding, misfolded protein binding, and PDI activity. These genes include *Xbp1*, *Hspa5*, *Dnajc3*, and members of the *Pdi* family, which overlap with *C. elegans* homologs in our transcriptomic dataset and are required for *xbp-1s-*mediated suppression of tauopathy. Additionally, overexpression of *Nr4a1* or *Hspa5* improved long-term memory in a tau-based mouse model of AD. Together with our findings, these results begin to describe a mechanism whereby upregulation of *xbp-1s* target genes can rescue neurological deficits.

Taken altogether, XBP-1s activates a network of genes involved in protein processing, lipid remodeling, carbohydrate metabolism, and apoptosis, all of which are required for *xbp-1s*-mediated suppression of tauopathy in *C. elegans* (Fig. 8). These data support a model where multiple *xbp-1s* target genes act in a UPR^ER^ transcriptional target network to ameliorate tauopathy. Among this network of target genes, *hsp-4/*BiP exhibits a prominent impact independent of other XBP-1s target genes that appears conserved across animal phyla as diverse as Nematoda and Mammalia. Given the intricate nature of XBP-1s-mediated transcriptional remodeling, future work should explore the functional interrelationships between UPR^ER^-regulated genes to mediate protection from pathological tau in disease. This will provide a deeper mechanistic understanding of XBP-1s-mediated neuroprotection in *C. elegans* tauopathy models and direct further studies in mammalian model systems, with the goal of modulating tauopathy in humans.

## Methods

### Plasmids

To generate transgenes expressing HSP-4 driven by a pan-neuronal *snb-1* promoter, a double stranded gene fragment of full-length *C. elegans hsp-4* was designed (gBlock Gene Fragment; Integrated DNA Technologies, Inc., Coralville, IA, USA). The *hsp-4* gBlock Gene Fragment served as a fragment for Gibson assembly^112^ using the *snb-1p* vector linearized with SacI and KpnI restriction enzymes.

### *C. elegans* strains and transgenics

*C. elegans* strains used are listed in Supplementary Table 3. All strains were maintained at 20 °C on standard nematode growth media (NGM) plates containing OP50 *Escherichia coli* (*E. coli*)^113^. To enhance animal yield for biochemical and molecular analysis, *C. elegans* were grown on NGM plates containing five times more peptone (NGM 5X PEP plates) prior to collection for protein and RNA extraction using established methods^31,114,115^. The *C. elegans* husbandry and experimentation was conducted in accordance with all relevant ethical and safety regulations for animal testing and research.

The *hsp-4* (high) and *hsp-4* (low) transgenic *C. elegans* strains were engineered by microinjection using a Nikon Eclipse TE300 microscope (Nikon Instruments, Inc., Melville, NY, USA) and Eppendorf FemtoJet® electronic microinjector setup (Eppendorf, Hamburg, Germany) into N2 (non-Tg) *C. elegans* at a concentration of either 100 ng/µl *snb-1p::hsp-4*; 20 ng/μl *myo-3p::mCherry*; 50 ng/μl *pUC19* carrier DNA or 50 ng/μl *snb-1p::hsp-4*; 30 ng/μl *myo-3p::mCherry*; 70 ng/μl *pUC19* carrier DNA to produce worms carrying extrachromosomal arrays. Day one adult animals from these generated lines were irradiated for 48 s with a UV lightsource in a Stratalinker© UV Crosslinker 1800 (Stratagene, San Diego, CA, USA), after two sequential autocrosslink warm-ups to integrate the extrachromosomal array into the genome. Irradiated worms were separated onto 150 mm NGM 5X PEP plates and hard starved for several weeks to enhance detection of transgene genomic integration events^36,58^. Starved populations were washed onto fresh plates and a portion of live, fluorescently marked animals were recovered to individual plates and screened for integration. Successfully integrated lines were screened by isolating individual worms with 100% transmission of the *myo-3p::mCherry* marker and outcrossed with N2 males at least two times. Custom loss of function alleles in Supplementary Table 3 were generated using CRISPR-Cas9 genome editing technology by introducing purified active recombinant Cas9 protein and synthetic CRISPR guide RNAs (gRNAs)^49,116^.

### *C. elegans* behavioral analysis

#### Manual swimming assay

*C. elegans* swimming behavior was assessed using established methods^36^. *C. elegans* were developmentally synchronized using a timed egg-lay and grown until all reached day one of adulthood at 20 °C. Individual developmentally synchronized animals were placed in a 10 µl M9 buffer droplet on a 10 well Teflon-printed glass slide and allowed to acclimate to a liquid environment for 10 s. One thrash was defined as a bend across the midline or two consecutive bends from the midline toward the same side. The number of thrashes were counted during a one minute period and averaged for each strain. At least three biological replicate assay sessions with at least 10 animals per assay session were analyzed for statistical significance. Behavioral analysis was conducted blinded to genotype to avoid biases. However, when the strains to be assayed were visibly distinguishable, blinding was not feasible. For comparison of two groups, an unpaired *t*-test, two-tailed, was used. For comparisons of three or more groups, a one-way analysis of variance (ANOVA), followed by Tukey’s multiple comparison post-test, was used.

#### Automated swimming assay

Supplementary data was acquired using an automated platform for measuring *C. elegans* swimming behavior^47^. *C. elegans* were developmentally synchronized using a timed egg-lay and grown until all reached day one of adulthood at 20 °C. Animals were moved to the assay room and allowed to acclimate for at least 30 min. NGM plates were flooded with 1 ml M9 buffer and transferred to 35 mm NGM video plates lacking OP50 *E. coli*. Animals acclimated to M9 buffer for 10 s prior to a one minute video recording. Videos were acquired using the WormLab platform (MBF Bioscience, Williston, VT, USA). After videos were taken, worm movement behavior was analyzed using the WormTracker software (MBF BioScience, version 2020.1.1). The frequency of body bends, defined as turns by the software, was quantified as follows. A turn was defined as a change in the body angle defined by the quarter points and midpoint of the animal that was at least 20 degrees positive or negative from a straight line. Animals that were tracked for less than 30 s were omitted from analysis. The total number of body bends was divided by the track duration to give the frequency of body bends per second. This was multiplied by 60 s to give the frequency of body bends per minute. At least three biological replicate assay sessions with roughly 15–60 animals per assay session were analyzed for statistical significance. For comparison of two groups, an unpaired *t*-test, two-tailed, was used. For comparisons of three or more groups, a one-way ANOVA, followed by Tukey’s multiple comparison post test, was used.

#### Radial locomotion assay

*C. elegans* locomotion was assessed using published methods^117^. *C. elegans* were developmentally synchronized using a timed egg-lay and grown until all reached day one of adulthood at 20 °C. Developmentally synchronized animals were placed at the center of a 100 mm NGM 5X PEP plate. Animals were allowed to move freely for 1 h, and the radial distance traveled from the start point was recorded as radial dispersion in mm. At least two biological replicate assay sessions with at least 10 animals per assay session were analyzed for statistical significance. For comparison of three groups, a one-way ANOVA, followed by Tukey’s multiple comparison post-test, was used.

### *C. elegans* protein extraction

Protein was extracted from *C. elegans* using a rigorous lysis approach^36^. *C. elegans* were synchronized using hypochlorite treatment and grown until adulthood at 20 °C on NGM 5X PEP plates. Synchronized adult *C. elegans* were washed off NGM 5X PEP plates in M9 buffer, washed an additional four times in M9 buffer to remove excess OP50 *E. coli*, pelleted by a one minute centrifugation at 800 × *g*, snap frozen in liquid nitrogen, and stored at −70 °C until protein extraction. A total of 2 µl of high-salt reassembly (RAB) buffer (0.1 M 2-(*N*-morpholino)ethanesulfonic acid, 1 mM ethylene glycol bis-2-aminoethyl ether-N,N’,N”,n’-tetraacetic acid, 0.5 mM MgSO_4_, 0.75 M NaCl, 0.02 M NaF, pH 7.0) containing phenylmethylsulfonyl fluoride and protease inhibitors was added per milligram of packed worm pellet wet weight and homogenized via sonication four times at 70% power for eight seconds. This total soluble worm lysate was reserved for subsequent immunoblotting.

### Protein immunoblotting

*C. elegans* protein immunoblotting was performed using Criterion apparatus (Bio-Rad) as recommended by the manufacturer (Bio-Rad Laboratories, Hercules, CA, USA)^36^. *C. elegans* protein preparations were diluted 1:5 (w/v) with sample buffer (0.046 M Tris, 0.005 M ethylenediaminetetraacetate, 0.2 M dithiothreitol, 50% sucrose, 5% sodium dodecyl sulfate, 0.05% bromophenol blue), sonicated for 15 s three times at 70% microtip power, boiled for 10 min, and centrifuged at 16,100 × *g* for 1–2 min. A total of 10 µl of lysate preparation (representing ~2 µg of protein) were loaded and resolved on precast 4 to 15% gradient SDS-PAGE gel and transferred to PVDF membrane as recommended by the manufacturer (Bio-Rad Laboratories, Hercules, CA, USA). PVDF membranes were blocked in 5% milk in PBS for 1 h before overnight incubation with primary antibody at 4 °C. The next day, PVDF membranes were washed in PBS with 0.1% Tween, incubated at room temperature with HRP-coupled secondary antibody for 2 h, and washed in PBS with 0.1% Tween before visualization. The dilutions and pertinent details for all primary and secondary antibodies used are listed in Supplementary Table 4. Enhanced chemiluminescence substrate (Bio-Rad Laboratories, Hercules, CA, USA) was added to the PVDF membrane, and chemiluminescence signals were detected with ChemiDocIt®^2^ 510 Imager (UVP LLC, Upland, CA, USA) or LiCor Odyssey Fc 2800 (LI-COR Biosciences, Lincoln, NE, USA). Relative intensity of chemiluminescence signals was measured with ImageJ Java^118^. At least three biological assay replicates were analyzed for statistical significance. For comparison of two groups, an unpaired *t*-test, two-tailed, was used. For comparison of three or more groups, a one-way ANOVA, followed by Tukey’s multiple comparison post-test, was used.

### *C. elegans* RNA purification

To achieve a tightly synchronized *C. elegans* population, two rounds of synchronization were completed. First, mixed population *C. elegans* were synchronized using hypochlorite treatment and grown until adulthood at 20 °C on NGM 5X PEP plates. From this adult population, *C. elegans* were synchronized using hypochlorite treatment and grown until L2 larval stage at 20 °C on NGM 5X PEP plates. Synchronized L2 *C. elegans* were washed off NGM 5X PEP plates in M9 buffer, washed an additional four times in M9 buffer to remove excess OP50 *E. coli*, pelleted by a one minute centrifugation at 800 × *g*, snap frozen in liquid nitrogen, and stored at −70 °C until RNA extraction. RNA was purified from snap-frozen packed *C. elegans* pellets using TRIzol Reagent as directed by the manufacturer’s instructions^54^. RNA was resuspended in 50 ul sterile water. RNA concentration and purity were assessed using a NanoPhotometer® NP80 spectrophotometer (Implen GmbH, Munich, Germany). RNA integrity was assessed by 1% TBE agarose gel electrophoresis.

### Library construction and transcriptomic analysis

*C. elegans* cDNA libraries for next generation sequencing were prepared from purified total RNA using the TruSeq reagents (Illumina, Inc., San Diego, CA, USA) at the Northwest Genomics Center within the University of Washington, Department of Genome Sciences. Libraries were sequenced using Illumina NovaSeq technology (Illumina, Inc., San Diego, CA, USA) with at least 25 million reads per sample. Three biological replicates with two technical replicates for each sample were analyzed. Transcriptomic analysis was completed using the Lasergene bioinformatics software version 17 (DNASTAR, Inc., Madison, WI, USA). The sequencing data was aligned to the *C. elegans* genome (release WB235) using Lasergene SeqMan NGen software (DNASTAR, Inc., Madison, WI, USA). Subsequent differential gene expression analysis was completed using Lasergene ArrayStar software (DNASTAR, Inc., Madison, WI, USA). Comparison of non-Tg and *xbp-1s* Tg animals yielded 2697 upregulated genes with two-fold or greater differential gene expression levels in *xbp-1s* Tg versus non-Tg animals. Among these genes, 560 genes exhibited significant gene expression changes in all four groups, analyzed by a two-way ANOVA, followed by a Benjamini-Hochberg post-test, with 131 of these genes exhibiting an *F*-value greater than 10. Filtering this gene set for genes with *xbp-1s* levels greater than 0.1 Reads Per Kilobase Million (RPKM) yielded a total of 116 significant genes in this analysis (Supplementary Table 1). The group of *xbp-1s* target genes was analyzed using the Basic Local Alignment Search Tool (BLAST)^119^ to identify human orthologs (BLAST expected values <1 × e^6^), yielding 11 significant genes (Table 1) and 5 significant genes with literature evidence of an association with ATF6 (Table 2). Gene Ontology (GO) term enrichment analysis was conducted using the Database for Annotation and Integrated Discovery (DAVID)^120^. Graphics representing analysis were generated using SRplot webtools^121^.

### Work with mice

Work with mice was reviewed and approved by the VA Puget Sound Health Care System Institutional Animal Care and Use Committee (IACUC) and conducted in an American Association for Accreditation of Laboratory Animal Care (AAALAC)-accredited animal research facility. We have complied with all relevant ethical regulations for animal use. C57BL/6J (non-Tg) was used as the control strain of mice, both sexes were used for study, and all strains used were maintained in a congenic state on this background. niXBP-1s mice were made by cross breeding two previously generated strains: the NEFH driver strain^86,87^ with the XBP-1s responder transgene^85^. For tissue harvest, mice were anesthetized and fixed by transcardial perfusion with 4% paraformaldehyde. Brains were removed and paraffin embedded for sectioning. Coronal sections (9 microns) were prepared and stored at 4 °C until use.

### Immunohistochemical evaluation

Animal brain sections were deparaffinized, rehydrated through alcohols, and processed through antigen retrieval steps consisting of heat pretreatment in citrate buffer by either microwave or autoclave per antibody-specific protocols. Sections were treated for endogenous peroxidases with 3% hydrogen peroxide for 30 min at room temperature, blocked in 5% non-fat milk in PBS for 1 h at room temperature, and incubated with primary antibodies overnight at 4 °C. Biotinylated secondary goat anti-mouse or goat anti-rabbit antibody was applied for 45 min at room temperature. The dilutions and pertinent details for all primary and secondary antibodies used are listed in Supplementary Table 4. Finally, sections were incubated in an avidin-biotin complex with streptavidin-HRP (Vector’s Vectastain Elite ABC-HRP kit, Burlingame, CA, USA) for 1 h at room temperature and the reaction product was visualized with 0.05% diaminobenzidine (DAB)/0.01% hydrogen peroxide in PBS. Negative controls consisted of full protocol except primary antibody. Digital images were obtained using a Leica DM6 microscope with a DFC 7000 digital camera (Leica Microsystems, Wetzlar, Germany) and imported into Adobe Photoshop (Adobe Inc, San Jose, CA, USA).

### Statistics and reproducibility

Statistical significance for all phenotype assays was determined using GraphPad Prism version 10.1.0 statistical software (GraphPad Software, Inc., Boston, MA, USA). Statistical significance is demarcated in figures as ns: *p* > 0.05, *p ≤ 0.05, **p ≤ 0.01, ***p ≤ 0.001, and ****p ≤ 0.0001. The details about experimental design and statistics used in different data analyses performed in this study are given in the respective sections of methods and figure legends.

### Schematic illustrations

Schematic illustrations in Figs. 1a, b, 7a and 8 were created using an academic license with the online application BioRender (Toronto, ON, Canada).

### Reporting summary

Further information on research design is available in the Nature Portfolio Reporting Summary linked to this article.

### Supplementary information

Supplementary Information

Description of Additional Supplementary Files

Supplementary Data 1

Reporting Summary

## Data Availability

The data analyzed for this study are published in this manuscript and associated Supplementary Information. The source data underlying the graphs in Figs. 2–6 and Supplementary Figs. 1–6, 8, 9 are available as a spreadsheet file called: Supplementary Data 1. The source data for uncropped full immunoblot images underlying Figs. 2b, e, 3b, 4b, f, j and Supplementary Figs. 6a, 7 are also available in Supplementary Fig. 10. The primary sequencing data and summary data files underlying Fig. 1, Tables 1, 2 and Supplementary Tables 1, 2 have been preserved (B.C.K. ORCID 0000-0002-2252-7634) as a DOI minted by Synapse.org: 10.7303/syn53060779.
