# Supplementary material for: Endoplasmic reticulum unfolded protein response transcriptional targets of XBP-1s mediate rescue from tauopathy
Source: Commun Biol. 2024 Jul 25;7:903. doi: 10.1038/s42003-024-06570-2 (PMC11282107; doi:10.1038/s42003-024-06570-2)
Supplement: Supplementary file 1 — Supplementary Information [file 42003_2024_6570_MOESM1_ESM.pdf]

Supplementary Table 1: XBP-1s responsive genes identified by RNAseq in *C. elegans*.

Worm Gene	RNA Fold Change		
	xbp-1s Tg vs. non-Tg	Tau (high) vs. non-Tg	Tau (high); *xbp-1s* Tg vs. Tau (high)
srbc-15	5882.456	0	0
srh-181	1155.178	4.327	782.717
srh-179	295.893	10.925	105.663
Y116F11B.9	251.487	-1.073	664.407
T18D3.14	125.641	1258.439	1.525
Y53G8AR.1	100.965	2.266	89.441
clec-241	97.237	0	34.518
ZK218.3	79.883	7.562	26.075
Y71A12B.8	50.274	3.316	51.270
irld-35	33.231	-6.106	3.854
C08F1.13	32.560	18.763	2.027
M04G7.5	32.282	-5.879	491.954
Y68A4A.12	31.429	22.300	3.190
fbxb-87	28.049	53.551	1.313
str-199	27.042	11.884	2.208
fbxa-170	26.705	36.269	2.587
F36D3.16	23.291	1.329	67.208
lipl-3	18.205	20.347	1.766
clec-209	17.775	2.236	-1.076
linc-64	17.558	20.083	3.296
F56A4.2	17.041	2.068	-1.106
F54H12.8	16.881	6.898	7.864
fbxb-53	16.789	4.023	8.010
K10G4.5	15.726	23.944	1.495
Y53G8B.3	15.614	7.966	2.221
F36A2.14	12.434	23.155	2.299
C44H9.4	10.979	9.040	4.429
csp-1	10.596	4.312	6.650
C04F12.1	10.040	1.748	8.173
ZK218.5	9.955	2.345	5.180
T20D4.12	7.424	-2.712	3.441
F42A6.3	6.713	-1.113	13.334
dnj-28	6.711	1.627	5.698
fbxa-185	5.825	9.050	2.137
F35F10.5	5.416	-1.888	3.265
F56A4.10	5.391	1.048	1.126
Y19D10A.8	5.335	-1.002	-1.029
F40G12.5	5.072	2.520	5.321
Y68A4A.13	5.021	12.496	1.044

C17B7.4	4.937	1.068	2.022
ZC239.5	4.844	1.858	2.805
C36B7.2	4.716	3.157	11.316
tsp-4	4.359	52.812	-5.421
hke-4.1	4.354	1.262	4.252
F41E7.6	4.315	-1.352	11.090
T20D4.10	4.257	-2.663	2.887
C09H10.4	4.163	1.100	4.482
C38D9.5	4.023	4.379	3.006
F42G8.7	3.980	-1.000	17.065
F35F10.7	3.954	1.115	2.284
F56A4.3	3.798	1.011	1.057
C17B7.15	3.600	-1.828	2.989
F36H5.10	3.579	2.882	1.727
Y19D10A.4	3.505	1.867	1.128
T28A11.25	3.405	1.064	1.703
C01B4.6	3.394	-1.002	1.337
F22E5.6	3.374	7.739	1.373
nhr-222	3.337	-1.172	6.549
F47H4.2	3.314	3.595	1.531
T20D4.11	3.312	-3.419	2.757
C17B7.12	3.292	-1.518	1.693
C38D9.2	3.279	4.260	1.478
sdz-35	3.254	10.259	-1.325
ZC404.15	3.220	1.413	3.377
B0041.11	3.152	1.300	4.629
acr-18	3.132	1.135	7.001
Y19D10A.5	3.116	1.319	-1.050
lys-10	3.098	7.183	1.737
H12D21.2	3.097	-2.005	7.438
ilys-3	3.082	9.152	1.100
F35F10.6	3.064	-1.137	1.511
Y19D10A.16	3.061	1.198	1.026
C17B7.2	3.054	-1.467	1.992
C36C5.5	3.004	-3.997	1.619
mct-2	2.917	1.716	-1.320
hsp-4	2.911	1.083	3.262
T20D4.20	2.902	-1.678	2.243
F41B4.3	2.865	1.467	3.336
T28A11.2	2.766	-1.220	1.965
C01B4.7	2.751	1.529	-1.059
C15H7.4	2.737	4.480	2.048
Y51B9A.8	2.689	-2.800	1.471

F56A4.12	2.684	1.208	-1.104
F40G12.6	2.676	1.344	4.637
K09F6.13	2.639	6.765	-1.219
C08F1.11	2.638	5.787	-1.099
clec-47	2.637	1.515	2.304
F17B5.1	2.600	1.789	1.624
K09F6.10	2.573	20.105	-2.478
C01B4.8	2.555	1.120	-1.04
Y45G12C.16	2.532	1.172	-1.108
C13B7.6	2.524	1.132	-1.038
Y82E9BR.1	2.486	2.708	1.145
Y38F1A.1	2.457	1.306	3.025
ZC412.9	2.433	-2.778	8.920
C08F1.8	2.420	3.405	1.412
F31E9.6	2.382	2.637	1.688
fbxa-52	2.378	3.578	1.462
F44G3.2	2.356	3.833	-1.251
ceh-83	2.307	4.201	1.822
F19G12.3	2.306	2.130	4.134
fbxa-189	2.271	4.395	-1.187
H36L18.2	2.269	-3.714	-1.087
Y71A12B.19	2.264	2.484	2.491
Y20C6A.1	2.235	3.795	-1.089
ZK218.1	2.232	2.412	2.814
rrf-2	2.228	3.487	1.543
ckb-2	2.151	1.539	1.948
K09C6.9	2.145	2.818	1.719
Y37H2A.16	2.140	3.401	1.019
Y43F8B.23	2.139	1.870	1.747
eol-1	2.034	2.899	-1.004
Y82E9BL.9	2.013	-2.353	-1.036
fbxa-11	2.009	1.865	1.301
nas-10	2.009	4.690	-1.671
C18D4.6	2.000	2.396	1.447

Supplementary Table 2: Neuronal overexpression of *xbp-1s* does not change tau mRNA detected by RNAseq.

Strain	Average Tau RPKM	Average *mec-12* RPKM	Tau/*mec-12* raw ratio
Tau (high)	632	222	2.8
Tau (high); ***xbp-1s*** Tg	686	280	2.4

The Tau (high) and Tau (high); ***xbp-1s*** Tg RNAseq datasets were re-assembled using the tau transgene mRNA sequence to determine whether changes in tau mRNA abundance might be caused by ***xbp-1s*** hyperactivity. Results were normalized by standard RPKM methods and by comparison to an alpha tubulin control comparator (***mec-12***). Methods were the same as the initial analysis (see Fig. 1).

Supplementary Table 3: *C. elegans* strains.

Abbreviation	Strain ID	Genotype	Variation Type	Genomic Change	Consequence	Paper Evidence	Outcrossed	Source
non-Tg	N2	Bristol, Great Britain wild type isolate	N/A	N/A	N/A	N/A	0x	CGC[1]
hsp-4 (high) Tg	CK1786	***snb-1p::hsp-4***; ***myo-3p::mCherry***	Transgenic	Random Integration	Multicopy Array	This Study	2x	This Study
hsp-4 (low) Tg	CK1911	***snb-1p::hsp-4***; ***myo-3p::mCherry***	Transgenic	Random Integration	Multicopy Array	This Study	3x	This Study
Tau (high)	CK144	***aex-3p::***hTau (4R1N); ***myo-2p::gfp***	Transgenic	Random Integration	Multicopy Array	[2,3]	2x	[2,3]
Tau (low)	CK1044	***aex-3p::***hTau (4R1N); ***myo-2p::gfp***	Transgenic	Random Integration	Multicopy Array	[2,3]	2x	[2,3]
xbp-1s Tg	AGD927	uthls270 [***rab-3p::xbp-1s; myo-2p::tdTomato***]	Transgenic	Random Integration	Multicopy Array	[4]	8x	[4]
ced-3 (-/-)	MT3002	***ced-3(n1286) IV***	Substitution	c/t (Wild Type/Variant)	Protein Change (W/*)	[5]	2x	CGC[1]
ckb-2 (-/-)	CK3106	***ckb-2(bk3106) III***	Deletion	0.9 kb Deletion	Exons 2-7 Deleted	This Study	2x	This Study
ckb-2 (-/-); ***xbp-1s*** Tg	CK3117	***ckb-2(bk3117) III; rab-3p::xbp-1s; myo-2p::tdTomato***	Deletion	0.9 kb Deletion	Exons 2-7 Deleted	This Study	2x	This Study
csp-1 (-/-)	RB1954	***csp-1(ok2570) II***	Insertion/Deletion	34 bp Insertion/1.5 kb Deletion	Splice Acceptor Variant	[6]	2x	CGC[1]
C01B4.6 (-/-)	tm6913	***C01B4.6(tm6913) V***	Deletion	0.64 kb Deletion	Splice Acceptor Variant	[6]	2x	NBRP[7]
dnj-27 (-/-)	RB1784	***dnj-27(ok2302) I***	Deletion	0.5 kb	Gene Deletion	[1]	2x	CGC[1]
dnj-28 (-/-) A	RB1917	***dnj-28(ok2490) I***	Deletion	2.1 kb Deletion	Splice Acceptor Variant	[6]	2x	CGC[1]
dnj-28 (-/-) B	CK3074	***dnj-28(bk3074) I***	Deletion	5.1 kb Deletion	Whole Gene Deletion	This Study	2x	This Study
eol-1 (-/-)	VC40248	***eol-1(gk534833) V***	Substitution	c/t (Wild Type/Variant)	Protein Change (W/*)	[8]	2x	CGC[1]
erp-44.3 (-/-)	tm6492	***erp-44.3/ceph-41(tm6492) IV***	Deletion	1.6 kb Deletion	Splice Acceptor Variant	[6]	2x	NBRP[7]
F41E7.6 (-/-)	tm5587	***F41E7.6/F41E7.7(tm5587) X***	Deletion	0.57 kb Deletion	Splice Acceptor Variant	[6]	2x	NBRP[7]
hsp-3 (-/-)	RB1104	***hsp-3(ok1083) X***	Deletion	1.4 kb Deletion	Splice Acceptor Variant	[6]	2x	CGC[1]
hsp-4 (-/-) A	VC1099	***hsp-4(gk514) II***	Deletion	0.75 kb Deletion	Splice Acceptor Variant	[6]	3x	CGC[1]
hsp-4 (-/-) B	CK3060	***hsp-4(bk3060) II***	Deletion	2.0 kb Deletion	Whole Gene Deletion	This Study	2x	This Study
lipl-3 (-/-)	VC40849	***lipl-3(gk846191) V***	Substitution	g/a (Wild Type/Variant)	Protein Change (Q/*)	[8]	2x	CGC[1]
mct-2 (-/-)	CK3105	***mct-2(bk3105) V***	Deletion	5.5 kb Deletion	Whole Gene Deletion	This Study	2x	This Study
Y19D10A.16 (-/-)	CK3109	***Y19D10A.16(bk3109) V***	Deletion	1.2 kb Deletion	Whole Gene Deletion	This Study	2x	This Study

Supplementary Table 4: Protein antibodies.

Antigen	Clone/ Product Name	Dilution	Host Species	Source	Catalog #
β-Tubulin	E7 mAb	1:5,000	Mouse	Developmental Studies Hybridoma Bank (Iowa City, IA, USA)	N/A
Tau (Total)	SP70 pAb	1:1,000	Rabbit	Rockland Immunochemicals Inc. (Limerick, PA, USA)	200-C01-B33
Tau (Total)	K9JA (DAKO) pAb	1:100,000	Rabbit	Agilent Technologies, Inc. (Santa Clara, CA, USA)	A0024
pTau Ser202	CP13 mAb	1:500	Mouse	Peter Davies (Litwin-Zucker Research Center for the Study of Alzheimer's Disease)	N/A
pTau Ser396/404	PHF-1 mAb	1:2,000	Mouse		
BiP/GRP78	4E3 mAb	1:1,000	Mouse	Thermo Fisher Scientific Inc. (Waltham, MA, USA)	MA5-15619
2º Ab Mouse	Horseradish Peroxidase α-Ms IgG (H+L)	1:5,000	Goat	Jackson Immunoresearch (West Grove, PA, USA)	115-035-146
2º Ab Rabbit	Horseradish Peroxidase α-Rb IgG (H+L)	1:5,000	Goat	Jackson Immunoresearch (West Grove, PA, USA)	111-035-144
XBP1s	D2C1F mAb	1:400	Rabbit	Cell Signaling Technology (Danvers, MA, USA)	12782
BiP/GRP78	4E3 mAb	1:200	Mouse	Thermo Fisher Scientific Inc. (Waltham, MA, USA)	MA5-15619
DNAJC3	J.98.6 mAb	1:250	Rabbit	Invitrogen (Waltham, MA, USA)	MA5-14820
2º Ab Mouse	α-Ms IgG (H+L), Biotinylated	1:200	Goat	Vector Laboratories, Inc. (Newark, CA, USA)	BA-9200
2º Ab Rabbit	α-Rb IgG (H+L), Biotinylated	1:200	Goat	Vector Laboratories, Inc. (Newark, CA, USA)	BA-1000

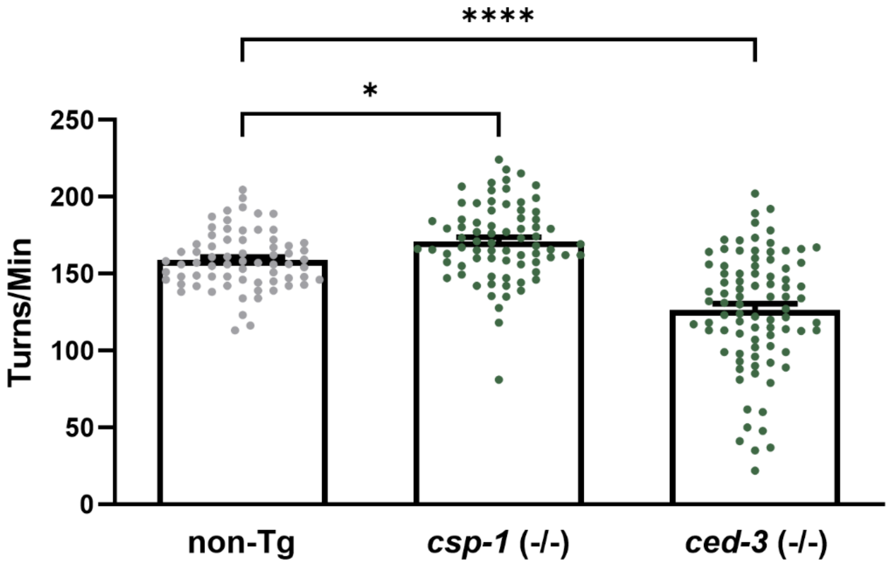

Supplementary Fig. 1: Loss of function of cytoplasmic resident caspases CSP-1 and CED-3 differentially affects locomotion in *C. elegans*. Loss of ***csp-1*** function causes mild behavioral enhancement, while loss of ***ced-3*** function causes mild behavioral defects observed as reflexive motor impairment in response to a liquid environment [***n*** = 69, 75, 87 animals, respectively; ***N*** = 3 biologically independent experiments; statistical analysis is by one-way ANOVA, followed by Tukey's post-test (*: ***p*** ≤ 0.05, ****: ***p*** ≤ 0.0001)]. Bar graph represents mean + SEM. Automated behavioral analysis was conducted essentially as described using the MBF WormLab system[9]. Source data are available as a Source Data file.

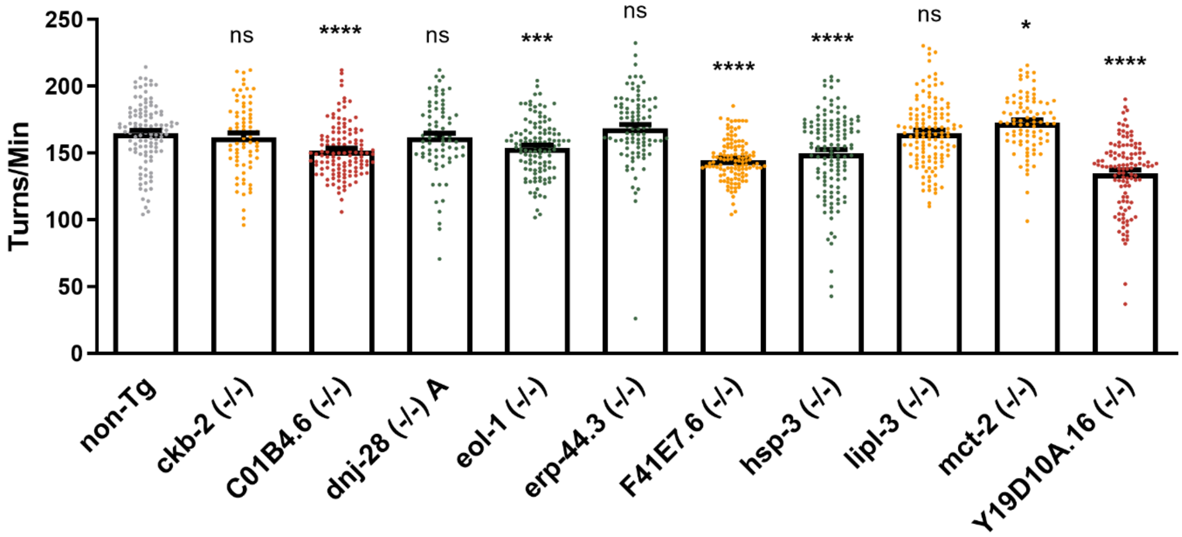

Supplementary Fig. 2: Loss of function of XBP-1s target genes differentially affects locomotion in *C. elegans*. Loss of function of ***ckb-2***, ***dnj-28***, ***erp-44.3***, and ***lipl-3*** does not affect behavior observed as reflexive motor impairment in response to a liquid environment. Loss of function of ***C01B4.6***, ***eol-1***, ***F41E7.6***, ***hsp-3***, and ***Y19D10A.16*** causes mild behavioral defects, while loss of ***mct-2*** function causes mild behavioral enhancement observed as reflexive motor impairment in response to a liquid environment [***n*** = 111, 72, 124, 75, 119, 93, 117, 129, 126, 85, 113 animals, respectively; ***N*** = 3 biologically independent experiments; statistical analysis is by unpaired ***t***-test, two tailed, comparing each loss of function gene to non-Tg (*: ***p*** ≤ 0.05, *****p*** ≤ 0.001, ****: ***p*** ≤ 0.0001)]. Bar graph represents mean + SEM. Automated behavioral analysis was conducted essentially as described using the MBF WormLab system[9]. Source data are available as a Source Data file.

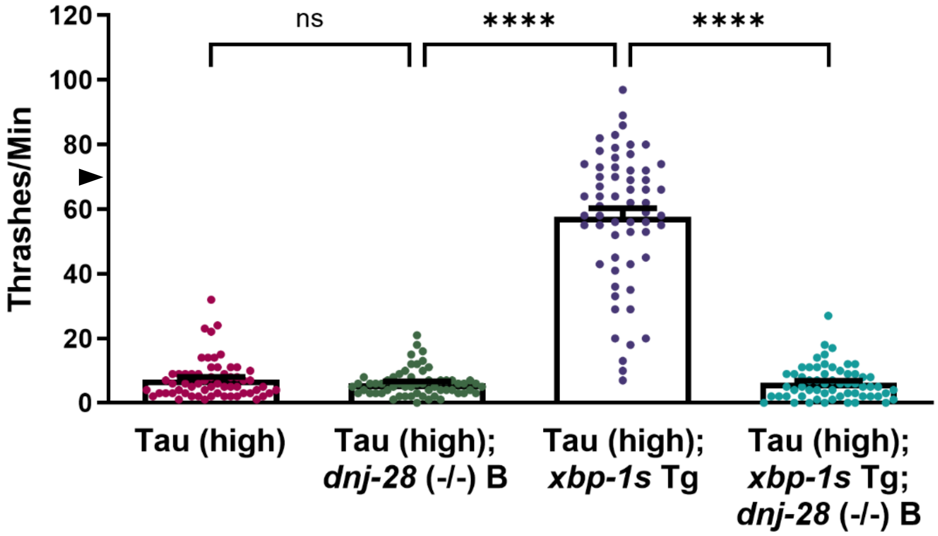

Supplementary Fig. 3: Loss of function of ER resident co-chaperone DNJ-28 using an independent allele is required for *xbp-1s*-mediated tauopathy suppression in transgenic *C. elegans*. Loss of ***dnj-28*** function abolishes the ability of neuronal overexpression of ***xbp-1s*** in Tau (high) animals to suppress severe behavioral defects observed as reflexive motor impairment in response to a liquid environment [***n*** = 60, 60, 60, 60 animals, respectively; ***N*** = 3 biologically independent experiments; statistical analysis is by one-way ANOVA, followed by Tukey's post-test (****: ***p*** ≤ 0.0001)]. Bar graph represents mean + SEM. Arrowhead on ***y***-axis denotes non-Tg animals average ~70 thrashes/min under standard laboratory conditions. Source data are available as a Source Data file.

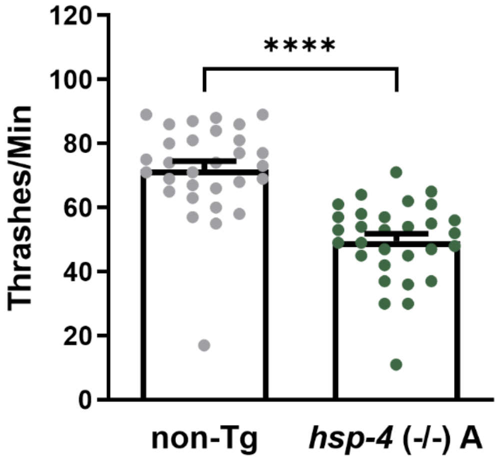

Supplementary Fig. 4: Loss of function of ER resident chaperone HSP-4 causes locomotion defects in *C. elegans*. Loss of ***hsp-4*** function causes mild behavioral defects observed as reflexive motor impairment in response to a liquid environment [***n*** = 30 animals; ***N*** = 2 biologically independent experiments; statistical analysis is by unpaired ***t***-test, two tailed (****: ***p*** ≤ 0.0001)]. Bar graph represents mean + SEM. Source data are available as a Source Data file.

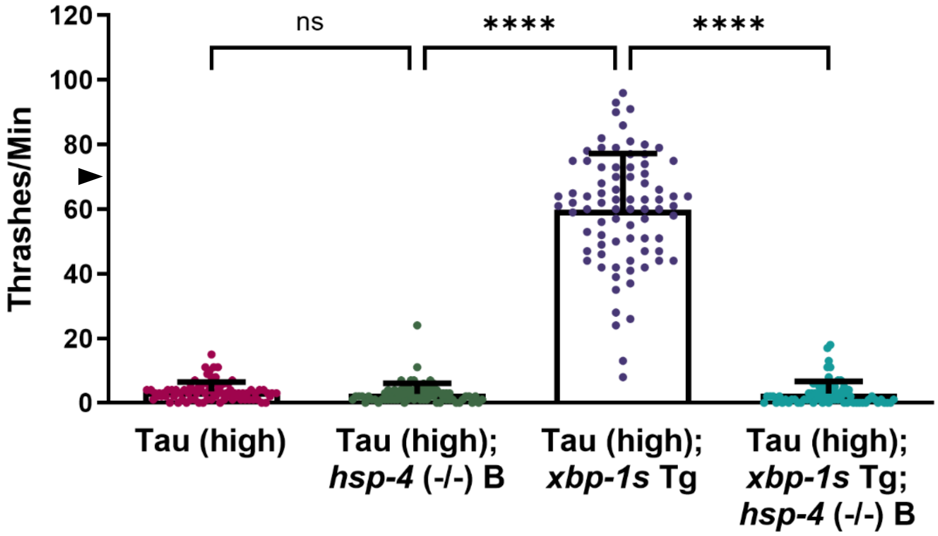

Supplementary Fig. 5: Loss of function of ER resident chaperone HSP-4 using an independent allele is required for *xbp-1s*-mediated tauopathy suppression in transgenic *C. elegans*. Loss of ***hsp-4*** function abolishes the ability of neuronal overexpression of ***xbp-1s*** in Tau (high) animals to suppress severe behavioral defects observed as reflexive motor impairment in response to a liquid environment [***n*** = 80, 75, 80, 74 animals, respectively; ***N*** = 4 biologically independent experiments; statistical analysis is by one-way ANOVA, followed by Tukey's post-test (****: ***p*** ≤ 0.0001)]. Bar graph represents mean + SEM. Arrowhead on ***y***-axis denotes non-Tg animals average ~70 thrashes/min under standard laboratory conditions. Source data are available as a Source Data file.

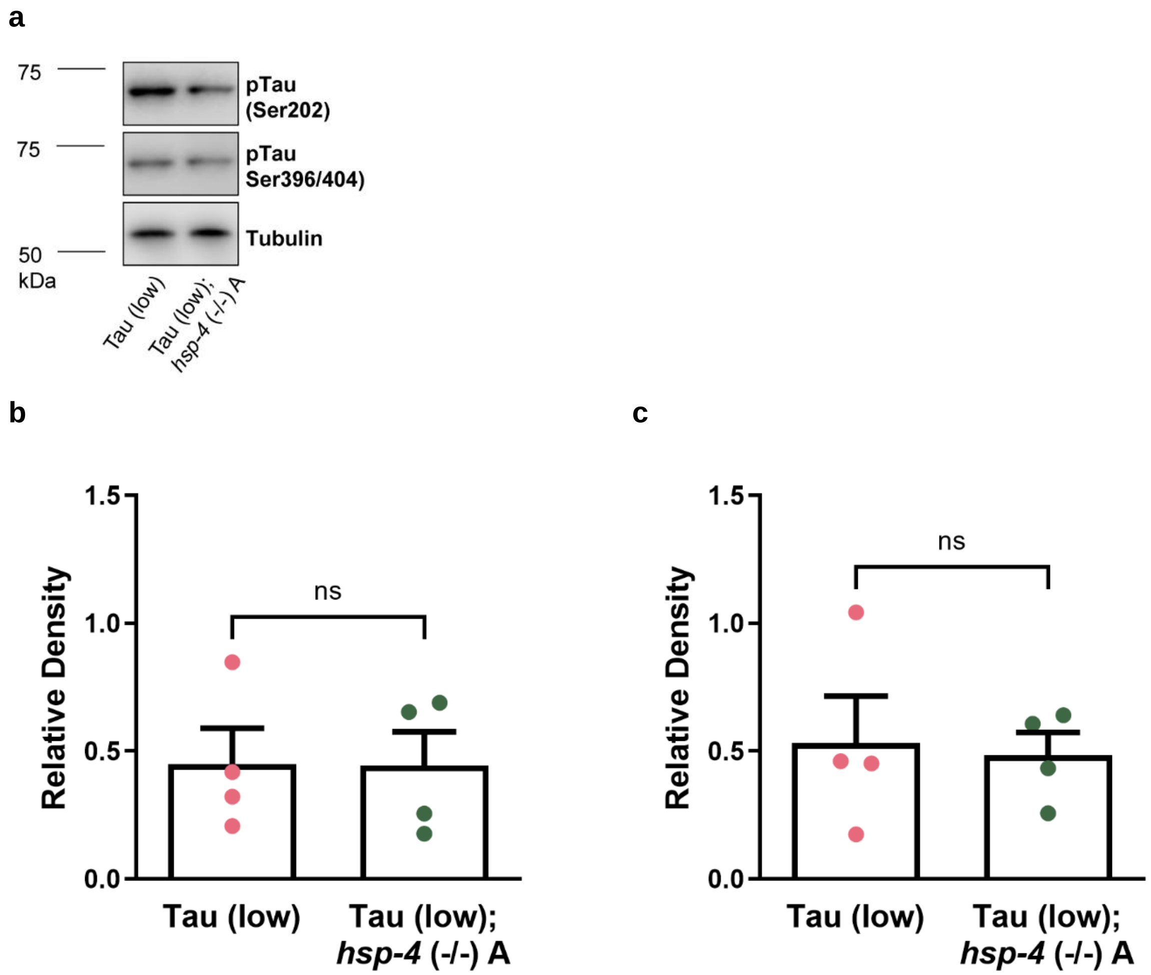

Supplementary Fig. 6: Loss of function of ER resident chaperone HSP-4 does not affect phosphorylated tau species in transgenic *C. elegans*. a Representative immunoblots for phosphorylated tau (Serine 202 and Serine 396/Serine 404 phosphorylation sites) and tubulin are shown. **b** Densitometry analysis of chemiluminescence signals for phosphorylated tau (Serine 202) normalized to tubulin are plotted [***N*** = 4 biologically independent experiments; statistical analysis is by unpaired ***t***-test, two-tailed (ns: ***p*** = 0.9794)]. **c** Densitometry analysis of chemiluminescence signals for phosphorylated tau (Serine 396/Serine404) normalized to tubulin are plotted [***N*** = 4 biologically independent experiments; statistical analysis is by unpaired ***t***-test, two-tailed (ns: ***p*** = 0.8200)]. Bar graphs represent mean + SEM. Source data are available as Source Data files.

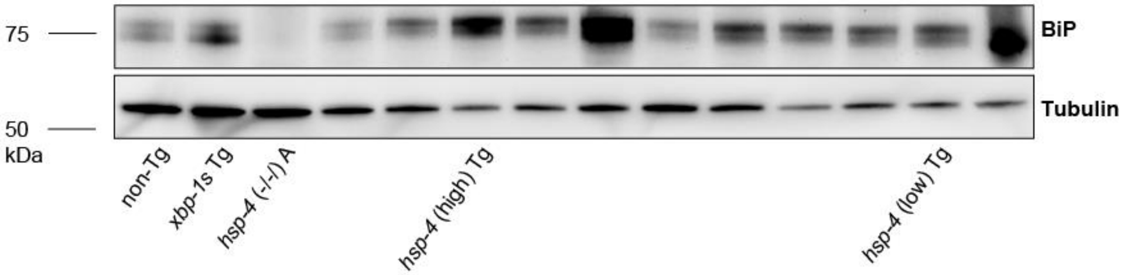

Supplementary Fig. 7: Pan-neuronal *hsp-4* transgenic *C. elegans* lines express varying levels of HSP-4/BiP. Immunoblots for HSP-4/BiP and tubulin are shown. To validate species cross-reactivity, ***xbp-1s*** Tg and ***hsp-4*** loss of function animals are included. ***hsp-4*** (low) Tg animals express approximately two-fold more HSP-4/BiP compared to non-Tg animals, and ***hsp-4*** (high) Tg animals express approximately greater than five-fold more HSP-4/BiP compared to non-Tg animals. Source data are available as a Source Data file.

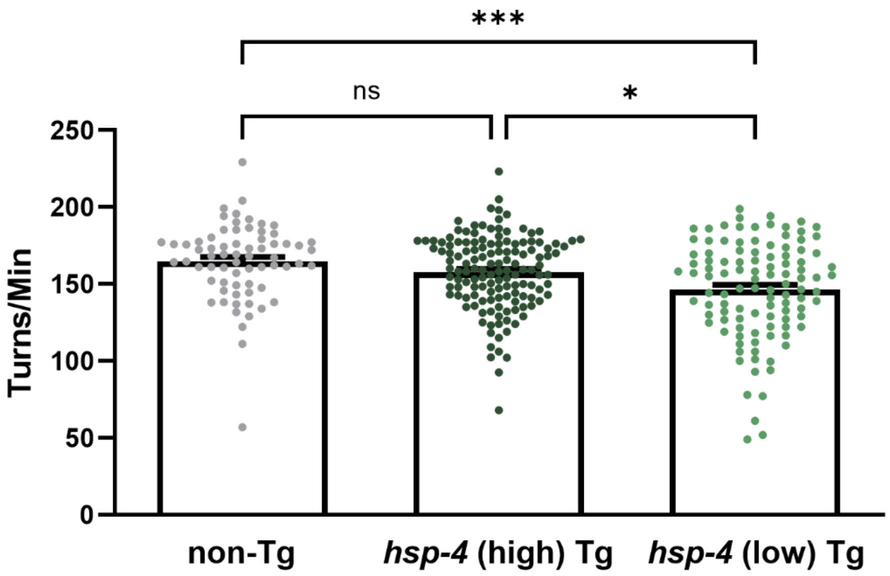

Supplementary Fig. 8: Neuronal overexpression of ER resident chaperone HSP-4 marginally impacts locomotion in *C. elegans*. Neuronal high overexpression of ***hsp-4*** does not affect behavior, while neuronal low overexpression of ***hsp-4*** causes mild behavioral defects observed as reflexive motor impairment in response to a liquid environment [***n*** = 68, 138, 107 animals, respectively; ***N*** = 3 biologically independent experiments; statistical analysis is by one-way ANOVA, followed by Tukey's post-test (***: ***p*** ≤ 0.001)]. Bar graph represents mean + SEM. Automated behavioral analysis was conducted essentially as described using the MBF WormLab system[9]. Source data are available as a Source Data file.

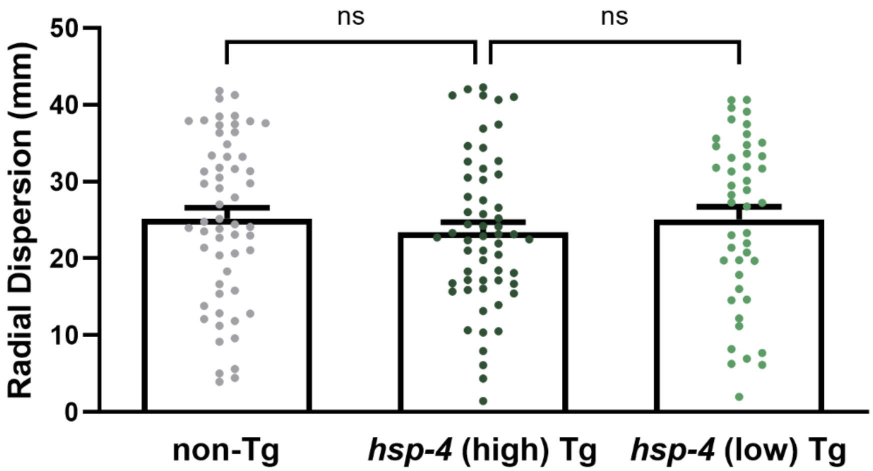

Supplementary Fig. 9: Neuronal overexpression of ER resident chaperone HSP-4 does not affect unstimulated locomotion in *C. elegans*. Neuronal high and low overexpression of ***hsp-4*** does not affect behavior observed in an unstimulated environment [***n*** = 57, 58, 45 animals, respectively; ***N*** = 3, 3, 2 biologically independent experiments, respectively; statistical analysis is by one-way ANOVA, followed by Tukey's post-test (ns: ***p*** = 0.6034)]. Bar graph represents mean + SEM. Source data are available as a Source Data file.

References

1 Stiernagle, T. Maintenance of C. elegans. *WormBook*, 1-11 (2006). https://doi.org/10.1895/wormbook.1.101.1

2 Waldherr, S. M., Strovas, T. J., Vadset, T. A., Liachko, N. F. & Kraemer, B. C. Constitutive XBP-1s-mediated activation of the endoplasmic reticulum unfolded protein response protects against pathological tau. *Nat Commun* **10**, 4443 (2019). https://doi.org/10.1038/s41467-019-12070-3

3 Taylor, L. M. *et al.* Pathological phosphorylation of tau and TDP-43 by TTBK1 and TTBK2 drives neurodegeneration. *Mol Neurodegener* **13**, 7 (2018). https://doi.org/10.1186/s13024-018-0237-9

4 Taylor, R. C. & Dillin, A. XBP-1 is a cell-nonautonomous regulator of stress resistance and longevity. *Cell* **153**, 1435-1447 (2013). https://doi.org/10.1016/j.cell.2013.05.042

5 Shaham, S., Reddien, P. W., Davies, B. & Horvitz, H. R. Mutational analysis of the Caenorhabditis elegans cell-death gene ced-3. *Genetics* **153**, 1655-1671 (1999). https://doi.org/10.1093/genetics/153.4.1655

6 Consortium, C. e. D. M. large-scale screening for targeted knockouts in the Caenorhabditis elegans genome. *G3 (Bethesda)* **2**, 1415-1425 (2012). https://doi.org/10.1534/g3.112.003830

7 Mitani, S. *Japan National Bioresource Project C. elegans*, <https://shigen.nig.ac.jp/c.elegans/> (

8 Thompson, O. *et al.* The million mutation project: a new approach to genetics in Caenorhabditis elegans. *Genome Res* **23**, 1749-1762 (2013). https://doi.org/10.1101/gr.157651.113

9 Kow, R. L., Black, A. H., Henderson, B. P. & Kraemer, B. C. Sut-6/NIPP1 modulates tau toxicity. *Human Molecular Genetics* **32**, 2292-2306 (2023). https://doi.org/10.1093/hmg/ddad049

Supplementary Fig. 10

Figure 2b

A	Tau (high)
B	Tau (high); ***csp-1*** (-/-)
C	Tau (high); ***xbp-1s*** Tg
D	Tau (high); ***xbp-1s*** Tg; ***csp-1*** (-/-)

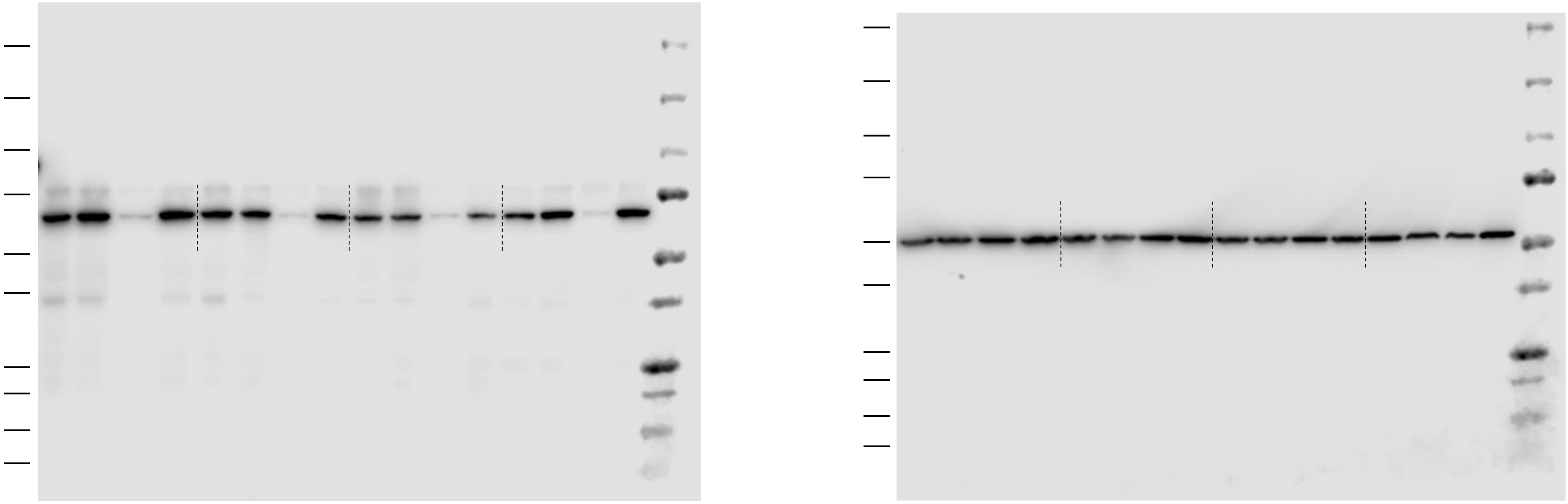

Total Tau (DAKO)

A B C D A B C D A B C D A B C D

250
150
100
75
50
37
25
20
15
10
kDa

Tubulin

A B C D A B C D A B C D A B C D

250
150
100
75
50
37
25
20
15
10
kDa

Figure 2e

A	Tau (high)
B	Tau (high); ***ced-3*** (-/-)
C	Tau (high); ***xbp-1s*** Tg
D	Tau (high); ***xbp-1s*** Tg; ***ced-3*** (-/-)

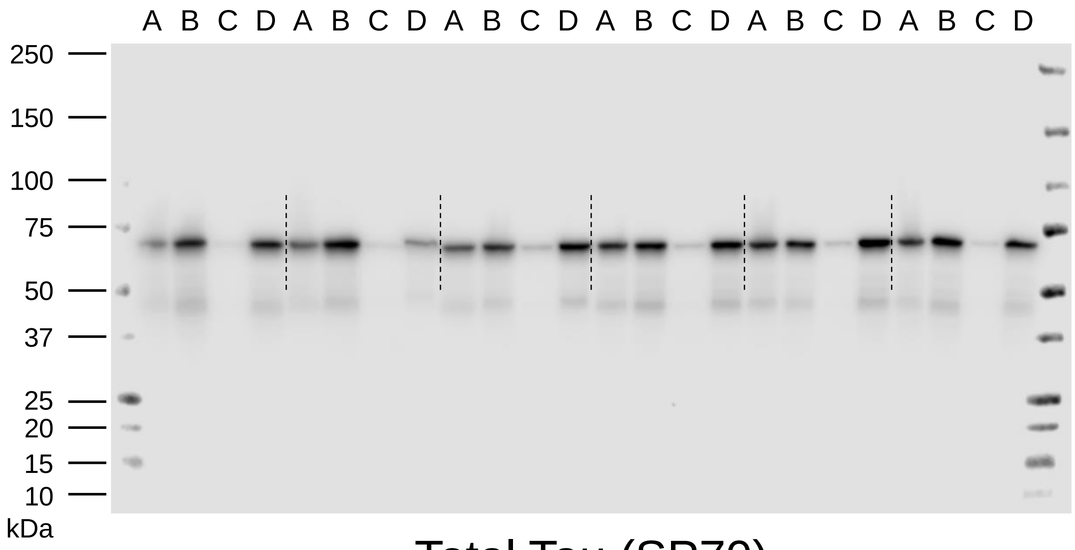

Total Tau (SP70)

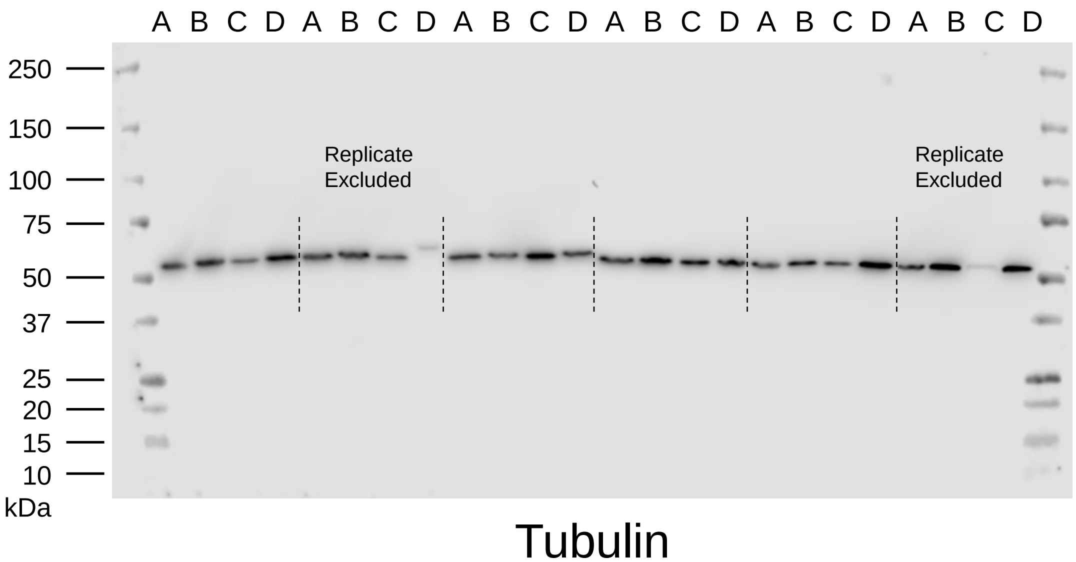

Tubulin

Figure 3b

A	Tau (high)
B	Tau (high); ***dnj-28*** (-/-) A
C	Tau (high); ***xbp-1s*** Tg
D	Tau (high); ***xbp-1s*** Tg; ***dnj-28*** (-/-) A

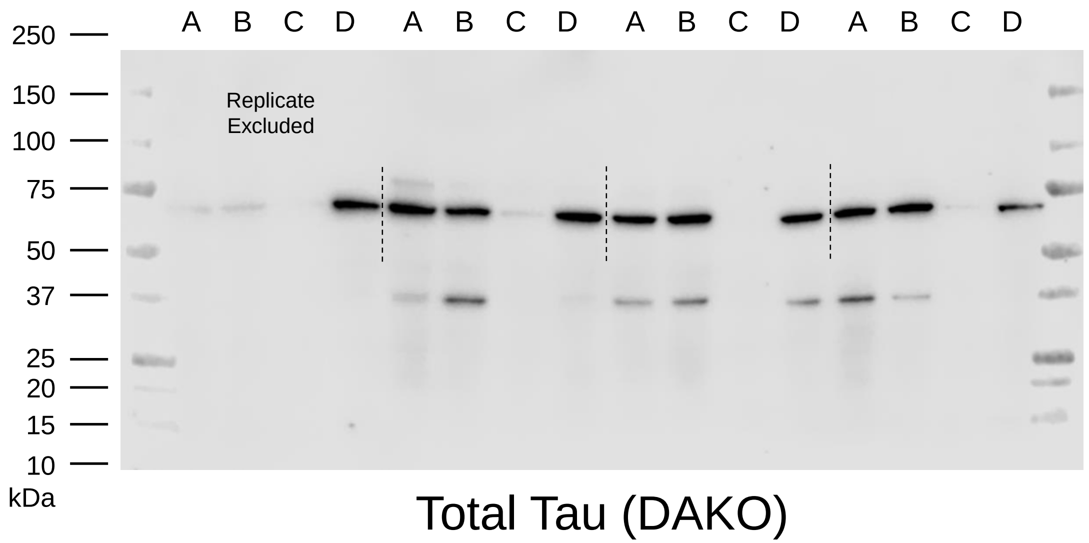

Total Tau (DAKO)

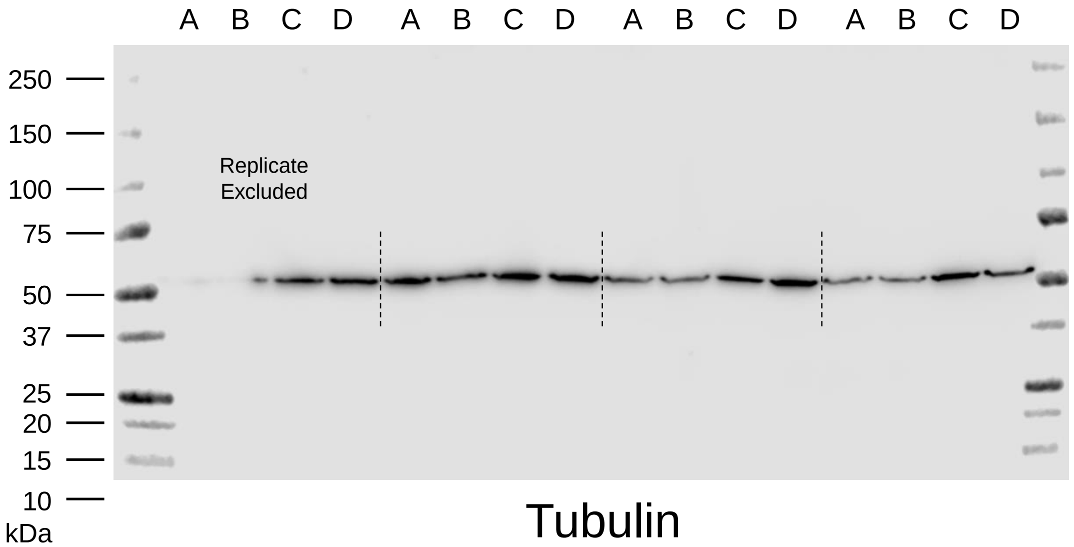

Tubulin

Figure 4b

A	Tau (high)
B	Tau (high); ***hsp-4*** (-/-) A
C	Tau (high); ***xbp-1s*** Tg
D	Tau (high); ***xbp-1s*** Tg; ***hsp-4*** (-/-) A

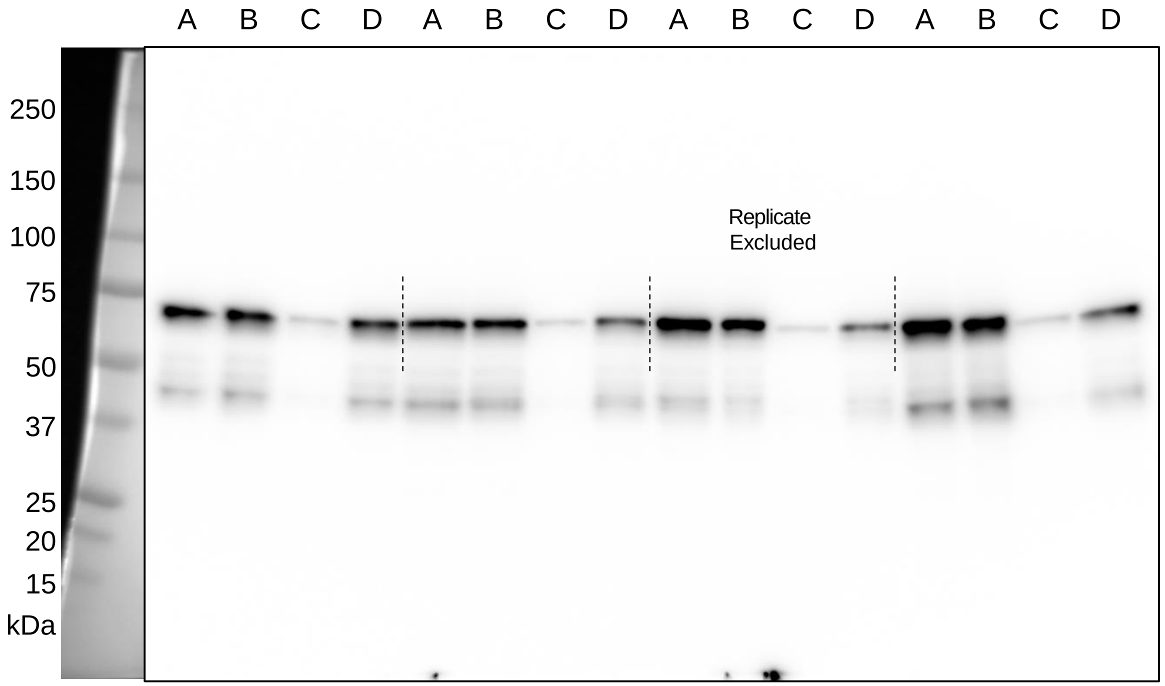

Total Tau (SP70)

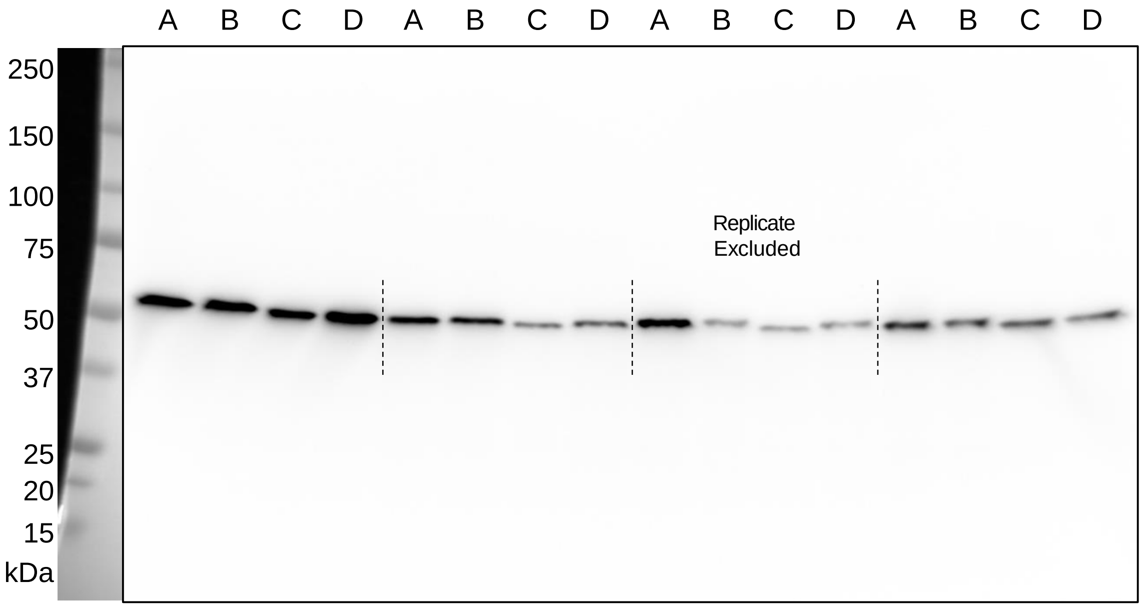

Tubulin

Figure 4f

A	Tau (low)
B	Tau (low); ***hsp-4*** (-/-) A

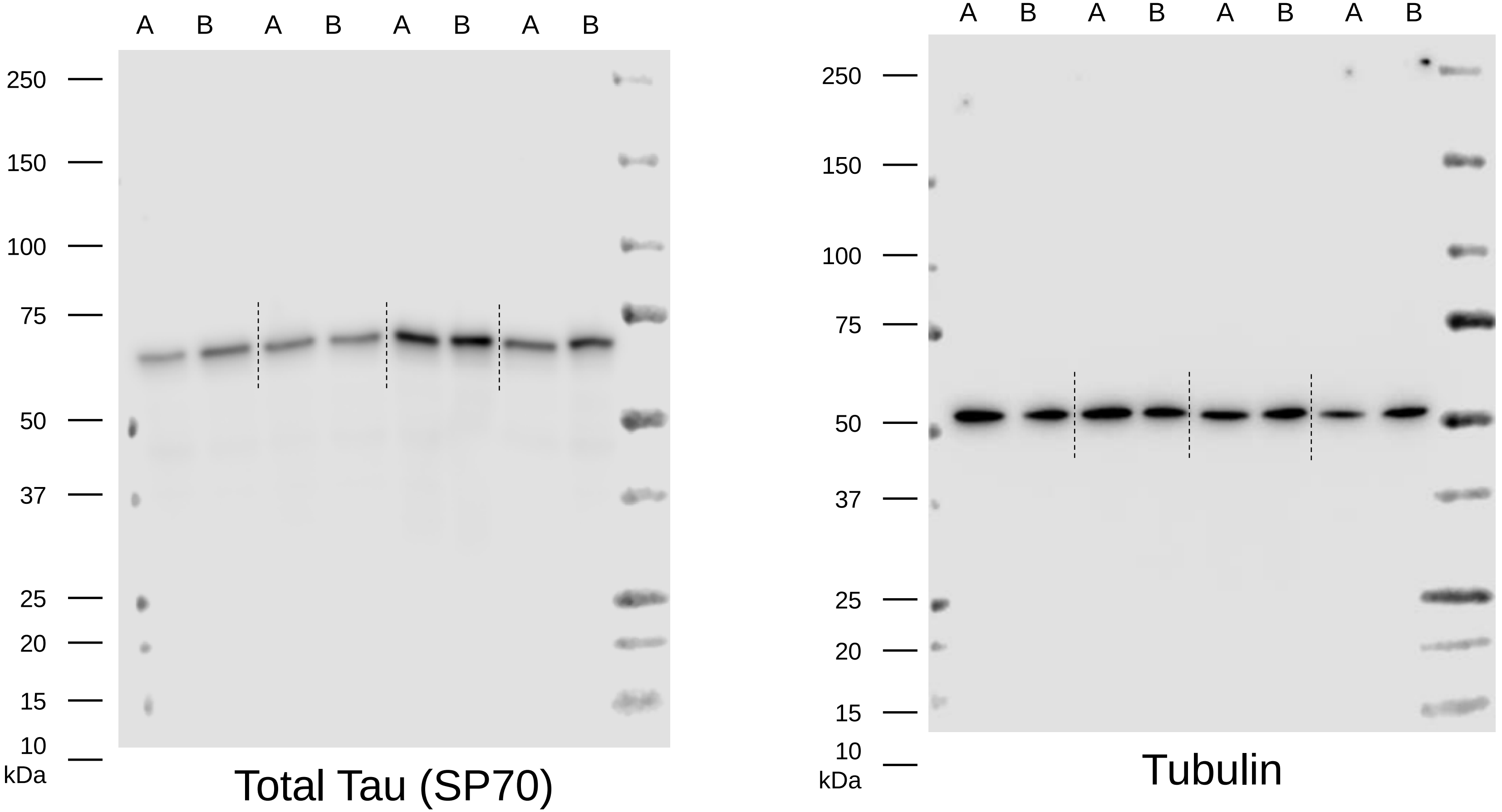

Figure 4j

A	Tau (high)
B	Tau (high); ***hsp-4*** (high) Tg
C	Tau (high); ***hsp-4*** (low) Tg

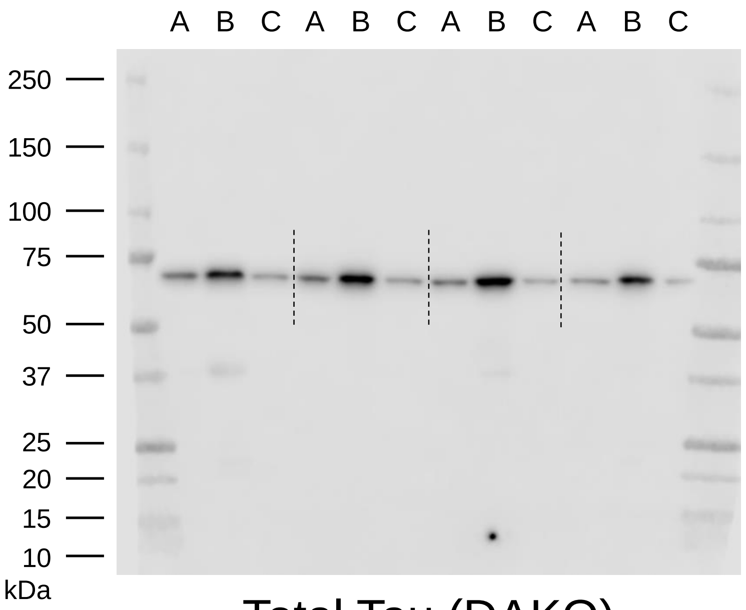

Total Tau (DAKO)

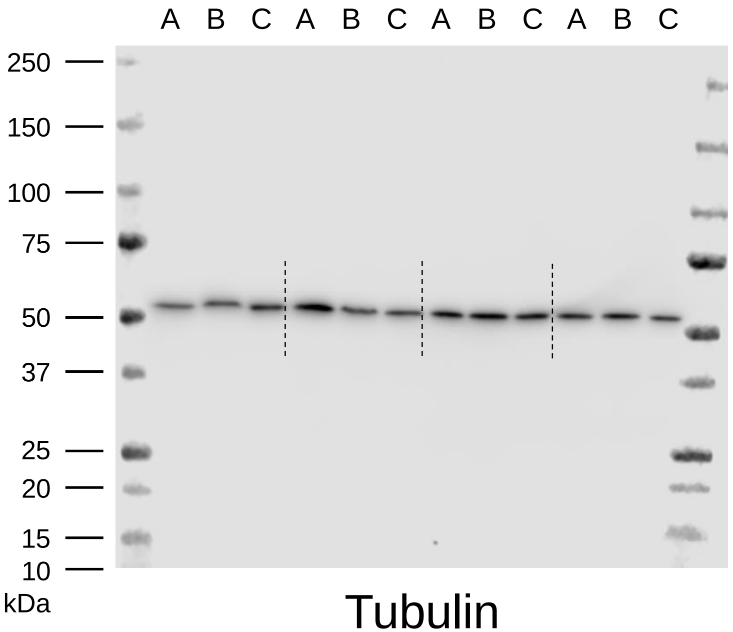

Tubulin

Supplementary Figure 6a

A	Tau (low)
B	Tau (low); ***hsp-4*** (-/-) A

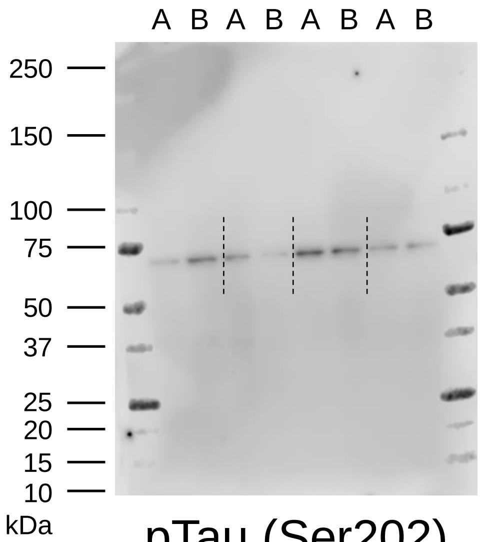

pTau (Ser202)

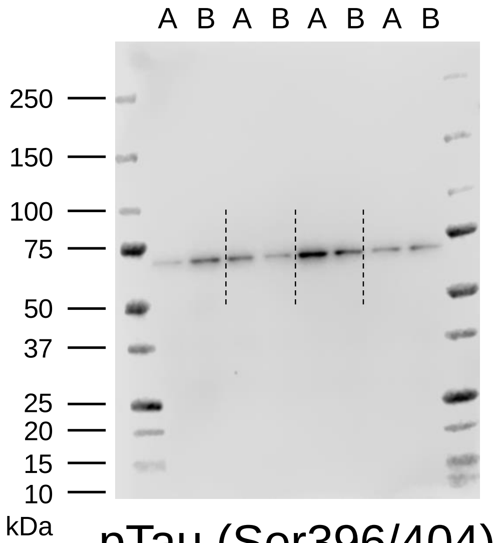

pTau (Ser396/404)

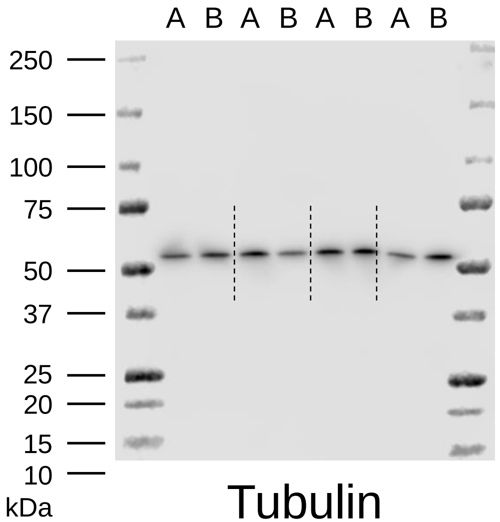

Tubulin

Supplementary Figure 7

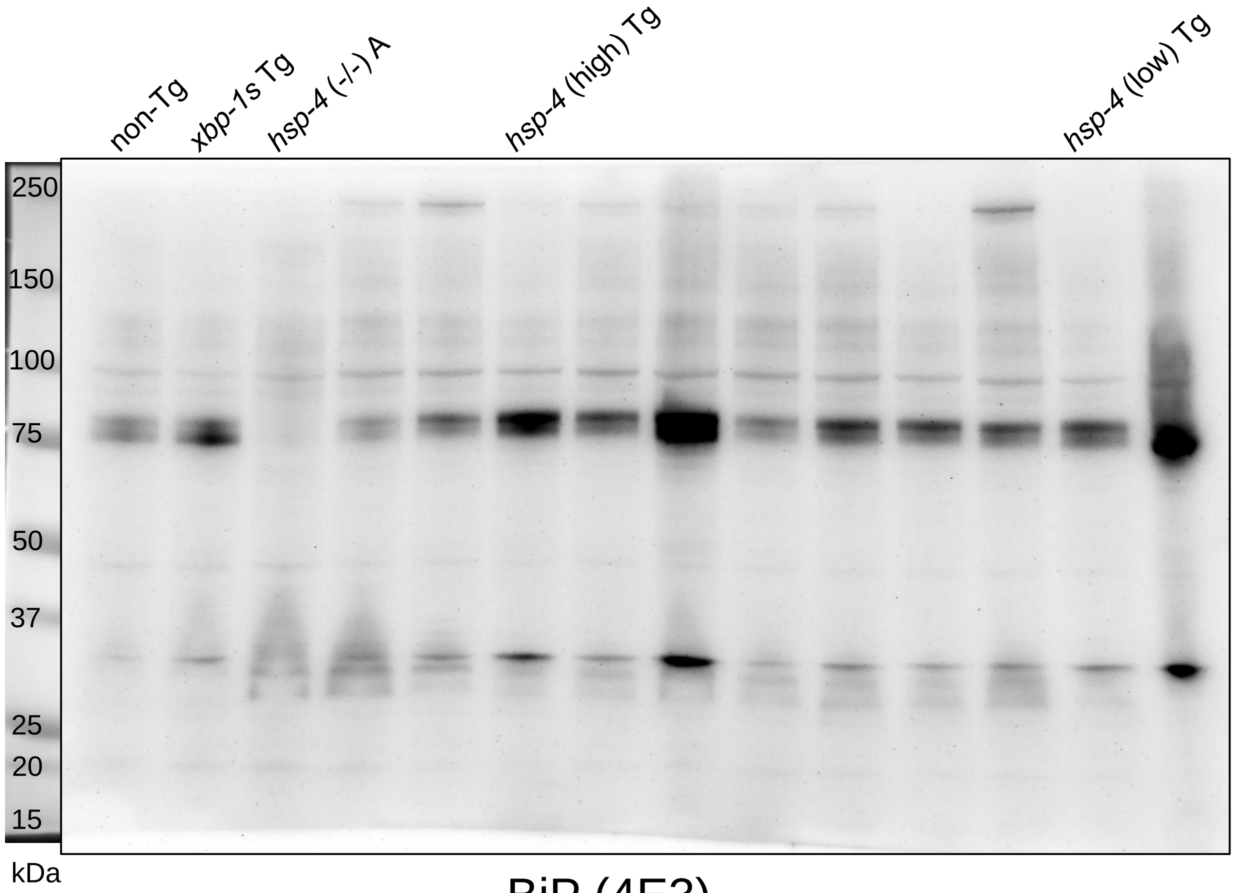

BiP (4E3)

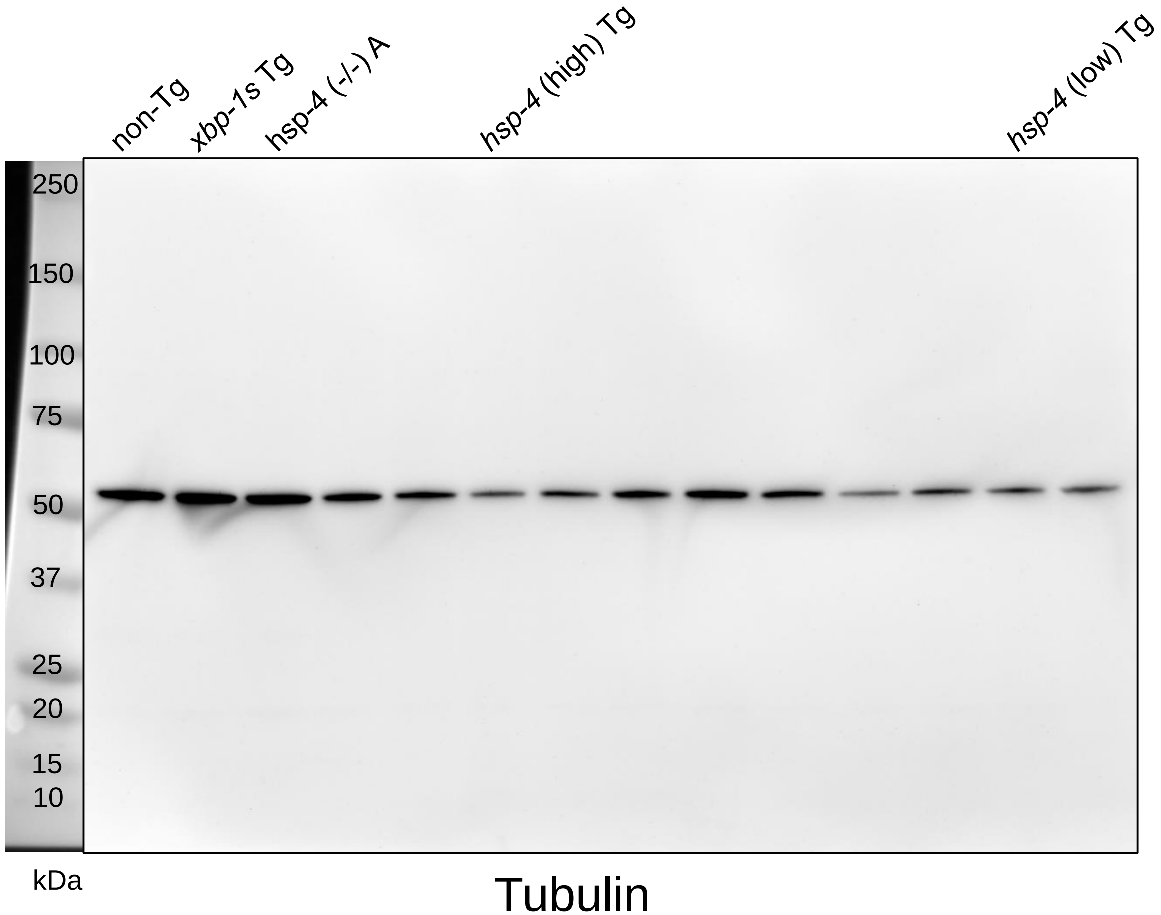

Tubulin
