# Supplementary material for: Endoplasmic reticulum unfolded protein response transcriptional targets of XBP-1s mediate rescue from tauopathy
Source: Commun Biol. 2024 Jul 25;7:903. doi: 10.1038/s42003-024-06570-2 (PMC11282107; doi:10.1038/s42003-024-06570-2)
Supplement: Supplementary file 2 — Description of Additional Supplementary Files [file 42003_2024_6570_MOESM2_ESM.pdf]

Description of Additional Supplementary Files:

File Name: Supplementary Data 1

Description: The source data underlying the graphs in Figs. 2-6 and Supplementary Figs. 1-6, 8-9 are available as a spreadsheet file called: Supplementary Data 1.
